# Revisiting macrophage metabolic reprogramming in metabolic dysfunction-associated steatotic liver disease: mechanisms, regulation, and therapeutic breakthroughs

**DOI:** 10.3389/fimmu.2026.1899587

**Published:** 2026-08-17

**Authors:** Li Zhu, Bo Wu

**Affiliations:** 1 Department of Pathology, The First Hospital of Jilin University, Changchun, China; 2 Department of Hepatobiliary and Pancreatic Surgery, General Surgery Center, The First Hospital of Jilin University, Changchun, China

**Keywords:** HIF-1α, macrophage metabolism, MASLD, metabolic therapy, TCA cycle

## Abstract

Metabolic dysfunction-associated steatotic liver disease (MASLD) affects over 30% of adults globally, yet therapeutic options remain limited. Macrophage metabolic reprogramming is increasingly recognized as an important contributor to disease progression. Hepatic macrophages from resident Kupffer cells (KCs) to infiltrating monocyte-derived macrophages (MoMFs) and triggering receptor expressed on myeloid cells 2 (TREM2) ^+^ lipid-associated macrophages (LAMs) shift their bioenergetic profile from fatty acid oxidation (FAO) and oxidative phosphorylation (OXPHOS) toward aerobic glycolysis. This review maps the metabolic circuits driving macrophage-mediated inflammation in MASLD. We delineate how tricarboxylic acid (TCA) cycle disruption generates signaling metabolites that enforce glycolytic commitment through hypoxia-inducible factor stabilization, how metabolic-epigenetic coupling perpetuates inflammatory programs, and how the failure of repair mechanisms and mitochondrial quality control accelerates tissue damage. Notably, the IRG1-itaconate axis exhibits a dynamic U-shaped trajectory across MASLD stages: itaconate levels decrease during early steatosis due to Kupffer cell loss, but subsequently rise markedly during MASH as infiltrating macrophages upregulate IRG1 expression, representing a compensatory yet insufficient anti-inflammatory response. We further examine how these metabolic states evolve across disease stages, from simple steatosis through steatohepatitis and fibrosis to cirrhosis, and assess the therapeutic potential of metabolic interventions. Recent FDA accelerated approvals of resmetirom and semaglutide for selected adults with non-cirrhotic MASH and F2-F3 fibrosis have expanded the therapeutic landscape. Their clinical benefits are primarily supported by histological endpoints; whether modulation of hepatic macrophage metabolism contributes directly to these benefits remains to be established. Emerging evidence indicates that macrophage metabolic states retain plasticity and can be pharmacologically reprogrammed, with single-cell metabolomics poised to guide precision therapeutic strategies.

## 1 Introduction

Metabolic dysfunction-associated steatotic liver disease (MASLD), formerly known as non-alcoholic fatty liver disease (NAFLD), has emerged as the most prevalent chronic liver condition globally. According to the Global Burden of Disease Study 2021, there were 1,267.9 million MASLD cases worldwide in 2021, with an age-standardized prevalence rate of 15,018.1 per 100,000 population, representing a 24.3% increase since 1990 (1). A recent comprehensive review further indicates that approximately 38% of all adults and 7%-14% of children and adolescents currently have MASLD, with projections suggesting that adult prevalence could exceed 55% by 2040 (2). The disease spectrum encompasses a continuum ranging from simple hepatic steatosis through metabolic dysfunction-associated steatohepatitis (MASH) to advanced fibrosis, cirrhosis, and ultimately hepatocellular carcinoma (HCC) (3). MASH was historically termed non-alcoholic steatohepatitis (NASH). Despite its enormous public health burden, therapeutic options for MASLD remain limited, with lifestyle modifications representing the cornerstone of current management strategies and only limited pharmacological interventions approved for clinical use (4).

The pathogenesis of MASLD is multifactorial, involving complex interactions between genetic predisposition, environmental factors, and metabolic derangements. Central to disease progression is the hepatic inflammatory response, orchestrated primarily by macrophages that populate the liver under both physiological and pathological conditions (5). The hepatic macrophage compartment comprises two major populations: resident Kupffer cells (KCs), which are embryonically derived and self-maintain under steady-state conditions, and monocyte-derived macrophages (MoMFs), which are recruited from the circulation in response to inflammatory signals and tissue injury (6). Both populations undergo profound phenotypic and functional alterations during MASLD progression, transitioning from homeostatic, anti-inflammatory cells to pro-inflammatory, tissue-damaging effectors (7).

A critical but historically underappreciated aspect of macrophage biology in MASLD is cellular metabolism. The recognition that immune cell function is intimately linked to metabolic programming, a field now termed immunometabolism, has revolutionized our understanding of inflammatory diseases (8). Homeostatic Kupffer cells are generally characterized by relatively preserved mitochondrial oxidative capacity and metabolic flexibility; however, their metabolic programs are heterogeneous and cannot be reduced to a simple fatty acid oxidation (FAO)/oxidative phosphorylation (OXPHOS)-versus-glycolysis dichotomy (9). These metabolic programs generate adenosine triphosphate (ATP) efficiently while minimizing the production of potentially harmful metabolic intermediates.

In MASLD, however, hepatic macrophages undergo profound metabolic reprogramming characterized by a shift away from oxidative metabolism toward aerobic glycolysis, a phenomenon reminiscent of the Warburg effect originally described in cancer cells (10). These changes enforced by hypoxia-inducible factor-1α (HIF-1α) stabilization and reinforced by epigenetic modifications, create a self-sustaining cycle of inflammatory activation and tissue damage. Concurrently, the disruption of the tricarboxylic acid (TCA) cycle at critical nodes generates signaling metabolites that amplify inflammation; and altered regulation of the IRG1-itaconate axis may weaken endogenous anti-inflammatory feedback in chronically activated hepatic macrophages. Recent evidence indicates that itaconate levels follow a dynamic U-shaped trajectory across MASLD stages: an initial decline during simple steatosis due to Kupffer cell loss is followed by a marked upregulation during MASH, driven by IRG1 induction in infiltrating monocyte-derived macrophages (reaching 400 ± 131 μM in murine WD-fed livers and 109 ± 9 μM in human NASH livers) (11–14). However, the direction and magnitude of itaconate changes across MASLD stages, macrophage subsets, and human tissues remain incompletely defined. The failure of repair programs mediated by specialized macrophage subsets, including triggering receptor expressed on myeloid cells 2 (TREM2) ^+^ lipid-associated macrophages (LAMs), further compounds these metabolic derangements (15). Given the central role of metabolic reprogramming in driving MASLD progression, there is intense interest in developing therapeutic strategies that target macrophage metabolism. Encouragingly, emerging evidence suggests that these metabolic states are not fixed but exhibit considerable plasticity (16). Peroxisome proliferator-activated receptor (PPAR) agonists, direct glycolytic inhibitors, and itaconate analogs have shown promise in preclinical models, demonstrating that metabolic reprogramming can be therapeutically targeted.

This review aims to provide a comprehensive analysis of hepatic macrophage metabolic circuits in MASLD pathogenesis, with two primary objectives: first, to map the metabolic transformation from fuel selection to mitochondrial dynamics, identifying critical nodes that drive macrophages toward pro-inflammatory, tissue-damaging phenotypes; and second, to assess whether these metabolic states are fixed or reversible, evaluating the evidence for metabolic therapies aimed at restoring macrophage homeostasis and halting disease progression. We will first delineate the heterogeneity of hepatic macrophage populations in MASLD (Section 2), then dissect the mechanistic underpinnings of metabolic reprogramming (Section 3), examine how these metabolic alterations evolve across disease stages (Section 4), and finally evaluate therapeutic opportunities that emerge from understanding macrophage metabolism in MASLD (Section 5).

## 2 Hepatic macrophage heterogeneity in MASLD

As the body’s largest metabolic and immune organ, the liver harbors a highly heterogeneous macrophage population comprising diverse subpopulations that differ in ontogeny, anatomical localization, and functional specialization. Recent advances in single-cell RNA sequencing (scRNA-seq) and spatial omics have profoundly reshaped our understanding of this cellular complexity. In the progression of MASLD, the hepatic macrophage compartment primarily encompasses resident KCs and MoMFs recruited from circulating monocytes, with the latter further differentiating into functionally distinct subsets, including LAMs and scar-associated macrophages (SAMs), thereby constituting a dynamic phenotypic continuum encompassing pro-inflammatory, pro-fibrotic, and metabolic regulatory properties (17–19). The detailed characteristics of each hepatic macrophage subset and their specific roles in MASLD will be discussed in the following sections. Hepatic macrophages have been increasingly recognized as central orchestrators of fatty liver disease pathogenesis, integrating metabolic, inflammatory, and fibrogenic signals across disease stages (20).

### 2.1 Resident Kupffer cells

KCs represent the largest and functionally central resident macrophage population in the liver, constituting over 90% of hepatic macrophages under physiological conditions. Embryonically derived from erythro-myeloid progenitors originating in the yolk sac, KCs seed the liver during early development and maintain population homeostasis through local self-renewal throughout adulthood, independent of bone marrow-derived monocyte input (21, 22). This ontogenic origin endows KCs with a unique tolerogenic phenotype, characterized by constitutive interleukin (IL)-10 and transforming growth factor (TGF)-β production and low expression of co-stimulatory molecules, thereby enabling the liver to maintain immune tolerance toward gut-derived antigens (23, 24).

Metabolically, KCs predominantly rely on FAO and OXPHOS to sustain their immunoregulatory functions, reflecting an intrinsic coupling between oxidative bioenergetics and tolerogenic phenotype in the hepatic microenvironment, as demonstrated in murine liver macrophages and validated in human liver tissue samples (25). The cell-intrinsic mechanisms underlying this oxidative-glycolytic phenotypic switch are examined in Section 3 from a pathway-centric perspective.

During MASLD progression, however, this homeostatic equilibrium is progressively disrupted: lipid overload and inflammatory stress induce endoplasmic reticulum stress and mitochondrial dysfunction in KCs, triggering their apoptotic loss and impairing self-renewal capacity (26). The resulting KC depletion creates a cellular niche that is rapidly occupied by monocyte-derived macrophages; yet these replacement cells lack the embryonic education of authentic KCs and fail to reconstitute tolerogenic functions, thereby contributing to the breakdown of hepatic immune homeostasis and amplification of meta-inflammation (27). Whether KC loss is a causative driver or a secondary consequence of MASLD pathogenesis remains an active area of investigation.

### 2.2 Monocyte-derived macrophages

As MASLD advances, the hepatic microenvironment undergoes profound remodeling, driving marked recruitment of peripheral Ly6C^hi^/C-C chemokine receptor type 2 (CCR2)^+^ classical monocytes into the liver under the direction of chemokine gradients, notably C-C motif chemokine ligand 2 (CCL2), where they differentiate into MoMFs. This monocyte-to-macrophage transition constitutes a critical amplification node in hepatic inflammation: damage-associated molecular patterns (DAMPs) and high-mobility group box 1 released by injured hepatocytes activate KCs, which in turn secrete CCL2, CCL3, and other chemoattractants, thereby establishing a self-sustaining feed-forward loop that perpetuates inflammatory monocyte influx (28).

Metabolically, MoMFs exhibit a glycolytic-dominant profile, contrasting with the oxidative metabolism of KCs. This metabolic characterization derives primarily from murine MASH models involving CCL2-driven monocyte recruitment, such as high-fat diet (HFD), as well as from *in vitro* polarized human monocyte-derived macrophages (hMDMs); direct metabolic profiling of hepatic MoMFs isolated from human MASLD livers remains technically challenging due to cell surface marker overlap with KCs (29).

Under conditions of extensive KC depletion (Section 2.1), a subset of MoMFs can progressively occupy the KC niche, acquiring KC-like marker expression such as Clec4F, and giving rise to monocyte-derived KCs (MoKCs). However, these cells frequently retain residual proinflammatory traits, with delayed or absent Tim4 expression, suggesting that their functional repertoire remains incomplete and not fully equivalent to that of embryonically derived KCs. Tran et al. demonstrated that in MASH models, MoKCs progressively supplant embryonic KCs; yet the former exhibit a heightened proinflammatory state coupled with diminished capacity to promote lipid metabolism, thereby exacerbating rather than ameliorating hepatic injury (30). These MoMFs and their derivative subsets subsequently differentiate into functionally specialized populations, including LAMs and SAMs, as detailed in the following sections.

### 2.3 Lipid-associated macrophages

#### 2.3.1 Origin, localization, and transcriptional identity

LAMs represent a characteristic macrophage subset in MASLD, initially identified in adipose tissue of obese individuals and subsequently recognized in the liver as context-dependent modulators of disease progression. Predominantly derived from infiltrating monocytes (Section 2.2), LAMs assemble into distinctive crown-like structures that encircle steatotic hepatocytes, a spatial arrangement revealed by spatial transcriptomics to be associated with local lipid handling and peri-hepatocellular inflammation propagation (31).

LAMs exhibit a distinctive transcriptional signature marked by robust expression of TREM2, CD9, GPNMB, SPP1, and FABP5, genes implicated in lipid uptake, phagocytic clearance, and extracellular matrix remodeling (19). Among these, TREM2 functions as a lipid-sensing receptor and serves as a master regulator of LAM identity. TREM2^+^ LAMs have context-dependent functions. They may support lipid handling and efferocytosis in some settings, whereas persistent lipid overload and inflammatory remodeling may impair their reparative capacity or promote profibrogenic interactions. We dissect this transition below.

#### 2.3.2 Protective phase: TREM2-mediated lipid sensing and efferocytosis

During the early stage of simple steatosis, hepatocyte-derived sphingosine-1-phosphate (S1P) induces TREM2 expression in LAMs, enabling efficient efferocytosis of lipid-laden apoptotic hepatocytes and thereby preserving local immune homeostasis (32). TREM2 recognizes phospholipids, apolipoproteins, and DAMPs on apoptotic hepatocyte surfaces, and signals through the adaptor protein DAP12 to promote macrophage survival, proliferation, and phagocytic function (33, 34). This positions TREM2^+^ LAMs as critical sentinels that limit lipotoxicity-driven inflammation. The metabolic mechanisms underlying TREM2-mediated metabolic regulation are discussed in Section 3.2.4.

#### 2.3.3 Functional collapse: ADAM17-mediated TREM2 shedding and efferocytosis failure

As MASLD progresses from simple steatosis to MASH, the hepatic microenvironment shifts toward a progressively proinflammatory milieu characterized by elevated tumor necrosis factor-alpha (TNF-α) and interleukin-1 beta (IL-1β). In murine MASH models, such as choline-deficient, L-amino acid-defined, high-fat diet (CDAHFD), and AMLN diet models, these cytokines activate nuclear factor kappa-light-chain-enhancer of activated B cells (NF-κB) signaling to upregulate ADAM metallopeptidase domain 17 (ADAM17) expression and activity (35, 36). Similar activation has been observed in human NASH liver tissue. ADAM17-mediated TREM2 shedding has been confirmed in both murine hepatic macrophages and human hepatic macrophage-like cells derived from tohoku hospital pediatrics-1 (THP-1), though the precise kinetics in primary human KCs require further validation.

ADAM17 mediates ectodomain shedding of TREM2, generating soluble TREM2 (sTREM2) and leaving a membrane-bound fragment that loses ligand-binding capacity. This mechanism was initially characterized in human Alzheimer’s disease microglia (37, 38) and subsequently validated in murine MASH hepatic macrophages (35); the extent to which identical cleavage kinetics operate in human MASLD hepatic macrophages remains an active area of investigation. Wang X et al. demonstrated that a TREM2 ectodomain-Fc fusion protein competitively inhibits ADAM17-mediated shedding, restores efferocytosis, and attenuates MASH progression in preclinical models (35). The metabolic consequences of TREM2 loss and the resulting metabolic-inflammatory coupling are detailed in Section 3.2.4.

#### 2.3.4 Consequences of uncleared apoptotic debris: inflammatory amplification

The loss of TREM2-dependent efferocytosis has profound pathological consequences. In murine MASH models (CDAHFD, Western diet) and human NASH explant tissue, uncleared apoptotic hepatocytes undergo secondary necrosis, releasing mtDNA, ATP, and high-mobility group box 1, potent DAMPs that activate pattern recognition receptors on neighboring macrophages and perpetuate inflammatory signaling (35).

This DAMP-driven activation recruits additional monocytes from the circulation, which differentiate into pro-inflammatory MoMFs that further exacerbate tissue damage (39). The resulting lipotoxic microenvironment from uncleared lipid-laden apoptotic cells feeds back to impair macrophage metabolism, establishing a self-sustaining metabolic-inflammatory vicious cycle that accelerates MASH progression (Section 3.2.4). Furthermore, the accumulation of cellular debris activates hepatic stellate cells, promoting myofibroblast transdifferentiation and collagen deposition (40, 41). This positions LAM dysfunction not only as a driver of inflammation but also as a priming event for fibrogenesis, bridging the transition to SAM-mediated scar formation (Section 2.4).

### 2.4 Scar-associated macrophages

SAMs represent a profibrogenic macrophage subset that emerges during advanced fibrosis, first identified through systematic single-cell sequencing of cirrhotic human liver by Ramachandran et al. (42). Predominantly residing within fibrous septa and scar tissue, these cells express a distinctive signature encompassing SPP1 (osteopontin), TREM2, CD9, and Lgals3 (galectin-3) (42). Although they share partial transcriptomic overlap with LAMs, SAMs are shaped by fibrotic microenvironments, including TGF-β-rich niches, toward profibrogenic transcriptional programs (25).

SAMs arise from infiltrating monocytes differentiating within the fibrotic microenvironment, independent of the KC lineage (5, 23). Functionally, SAMs display a pronounced profibrogenic phenotype: they directly activate hepatic stellate cells through secretion of TGF-β, platelet-derived growth factor, and connective tissue growth factor (27), thereby promoting myofibroblast transdifferentiation and collagen deposition. SAM expansion is strongly associated with disease severity. In liver tissue from MASH patients, the proportion of SAMs increases significantly with rising fibrosis stage (43). Furthermore, SAMs express proangiogenic factors that contribute to pathological vascular remodeling within fibrotic regions (42). TGF-β signaling plays a pivotal role in SAM differentiation and maintenance. Therapeutic blockade of TGF-β significantly reduces SAM abundance and attenuates hepatic fibrosis in preclinical models, establishing the TGF-β-SAM axis as a promising target for antifibrotic intervention (44).

### 2.5 Other transitional subsets and phenotypic continuum

In addition to these subsets, the MASLD liver harbors a spectrum of transitional or hybrid-phenotype macrophages. Proliferating macrophages (Ki67^+^) emerge during peak inflammatory phases, reflecting the local expansion demands of the macrophage compartment, whereas perivascular macrophages are distributed around central and portal veins, potentially contributing to vascular homeostasis (45). More importantly, scRNA-seq studies have revealed that the boundary between KCs and MoMFs is not sharply demarcated; rather, intermediate cells with hybrid phenotypes exist along a continuous spectrum. Notable among these are Clec4F^+^Tim4^–^ monocyte-derived KCs (pre-MoKCs), which progressively acquire KC characteristics while retaining residual expression of inflammatory genes (46).

**Figure 1:**
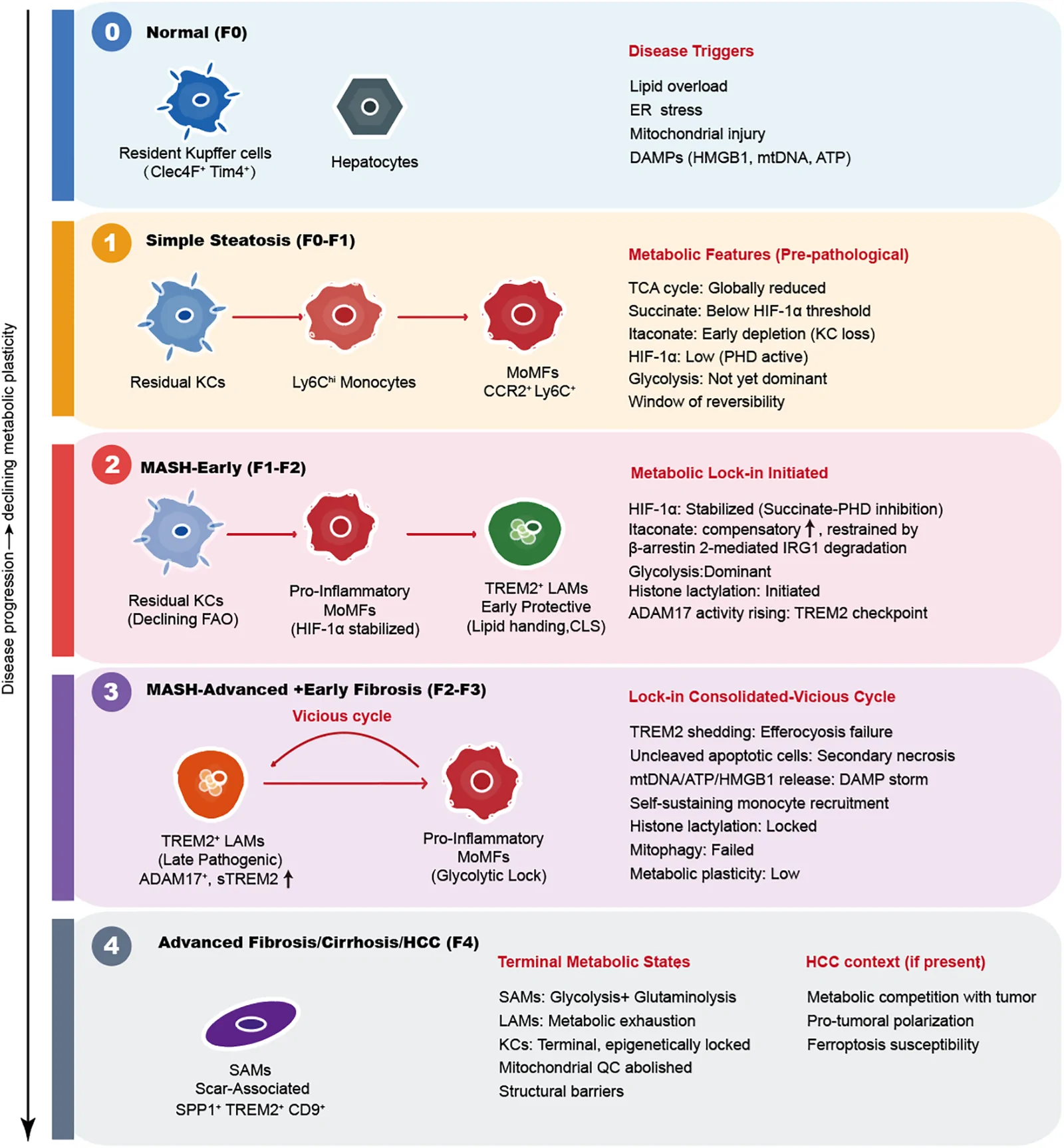
Metabolic reprogramming in hepatic macrophages during MASLD progression. Schematic depicting the transition of the hepatic macrophage compartment from the resident state through simple steatosis (F0-F1), early and advanced MASH (F1-F3), to advanced fibrosis/cirrhosis/HCC (F4). Disease triggers (lipid overload, ER stress, mitochondrial injury, DAMPs) drive KC decline, Ly6C^hi^ monocyte recruitment, and TREM2^+^ LAM accumulation. Metabolic lock-in occurs at early MASH, characterized by HIF-1α stabilization, succinate/prolyl hydroxylase (PHD) inhibition, compensatory but restrained itaconate elevation, glycolytic dominance, incipient histone lactylation; these changes are consolidated in advanced MASH (TREM2 shedding, efferocytosis failure, DAMP-driven vicious cycle, failed mitophagy). Terminal stages are marked by SAM expansion, metabolic exhaustion, epigenetic locking, and structural barriers to reversibility.

Collectively, these hepatic macrophage subsets, resident KCs, infiltrating MoMFs, TREM2^+^ LAMs, and profibrogenic SAMs, constitute a dynamic phenotypic continuum that undergoes profound metabolic reprogramming during MASLD progression (**Figure 1**).

## 3 Metabolic reprogramming mechanisms

### 3.1 TCA cycle disruption and the rise of signaling metabolites

**Figure 2:**
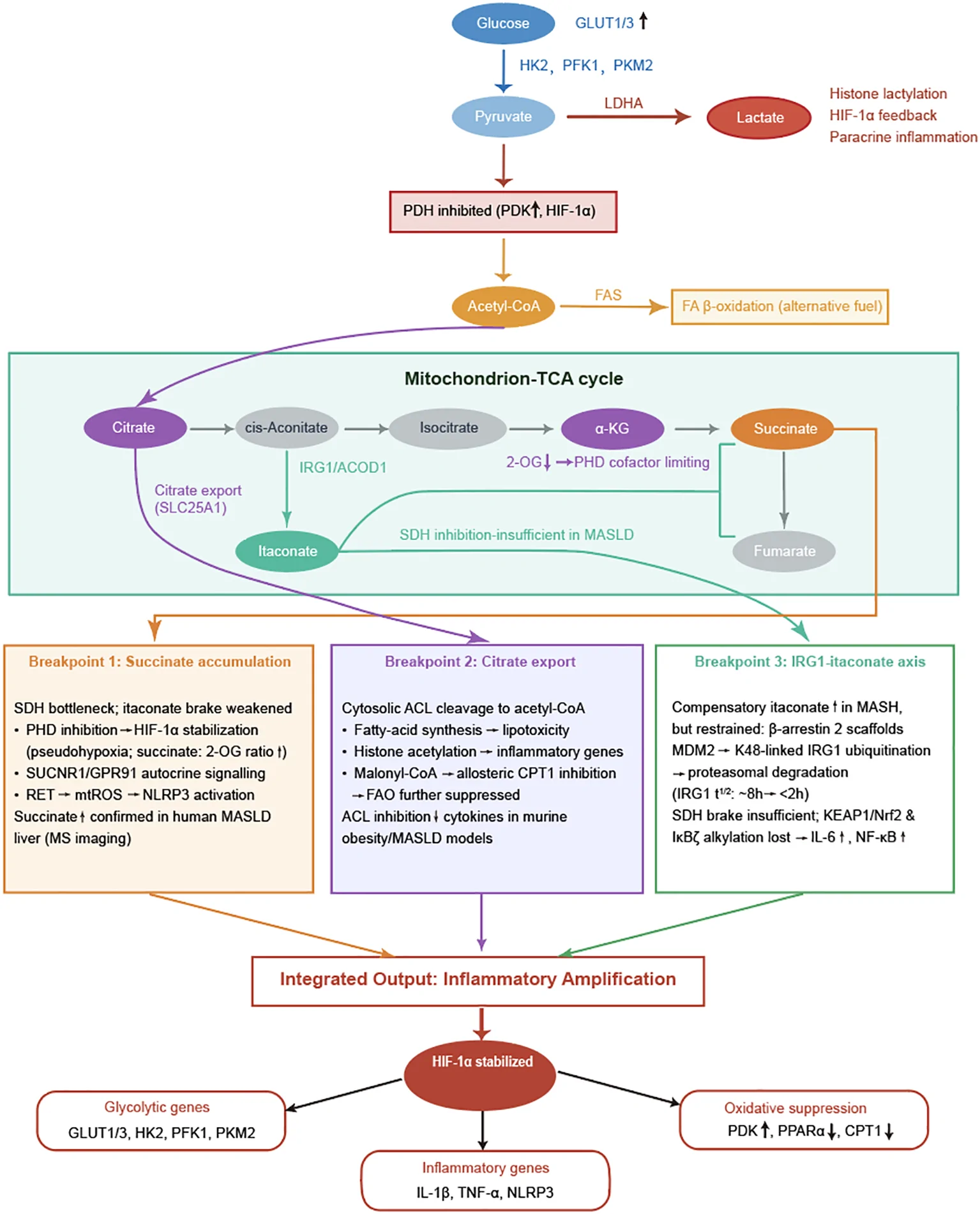
TCA cycle rerouting and signaling metabolites in MASLD-associated macrophages. Three breakpoints create a self-amplifying inflammatory network: (1) succinate accumulation upstream of the SDH bottleneck stabilizes HIF-1α via PHD inhibition and fuels mtROS production and succinate receptor 1 (SUCNR1)/G Protein-Coupled Receptor 91 (GPR91) signaling; (2) citrate export via ATP-citrate lyase (ACL) generates acetyl-CoA for lipogenesis and histone acetylation, while malonyl-CoA suppresses carnitine palmitoyltransferase 1 (CPT1) -dependent FAO; (3) the IRG1-itaconate axis undergoes a U-shaped, stage-dependent dysregulation: compensatory itaconate elevation in MASH is restrained by β-arrestin 2-scaffolded, MDM2-mediated K48 ubiquitination of IRG1 (half-life reduced from ~8 h → < 2 h), leaving the SDH brake insufficient. Converging on HIF-1α, these breakpoints lock in glycolysis and amplify inflammatory gene transcription, which encompasses IL-1β, TNF-α, and NOD-like receptor protein 3 (NLRP3), perpetuating macrophage-mediated liver inflammation.

Beyond the binary switch between OXPHOS and glycolysis lies a more nuanced disruption of the TCA cycle. Evidence from inflammatory macrophage models, murine MASH models, and transcriptomic studies suggests that TCA-cycle remodeling may contribute to macrophage activation in MASLD; however, direct flux-based evidence in primary human hepatic macrophages remains limited (**Figure 2**; **Table 1**):

**Table 1:** Role of key metabolites.

Metabolite	Origin/pathway	MASLD change	Mechanistic role(s)	Net effect on inflammation
Succinate	TCA intermediate; SDH breakpoint product	**↑↑**	Product inhibition of 2-OG-dependent dioxygenases (PHDs, histone demethylases) → HIF-1α stabilization; SUCNR1/GPR91 signaling; mtROS production	Pro-inflammatory
Itaconate	TCA-derived; IRG1/ACOD1 product	U-shaped: ↓ in steatosis → ↑↑ in MASH (infiltrating MoMFs)	SDH inhibition; kelch-like ech-associated protein 1 alkylation → Nrf2; IκBζ modification; Glycolysis suppression; Compensatory elevation in MASH counter-regulated by β-arrestin 2-mediated IRG1 degradation	Anti-inflammatory; compensatory response insufficient-augmentable by cell-permeable derivatives (4-OI, DI) in preclinical models
Lactate	Glycolytic end product	**↑↑**	Histone lactylation (p300/CBP); Paracrine inflammatory signal; HIF-1α stabilization feedback	Pro-inflammatory (epigenetic)
Citrate	TCA intermediate; ACL substrate	**↑**	Cytosolic export → acetyl-CoA; Fatty acid synthesis; Histone acetylation; Malonyl-CoA → CPT1 inhibition	Pro-inflammatory/Lipogenic
Acetyl-CoA	Central carbon metabolite	**↑**	Histone acetylation; Fatty acid synthesis; TCA cycle fuel (when PDH active)	Epigenetic + Metabolic
Malonyl-CoA	ACC product	**↑**	Allosteric CPT1 inhibition; Fatty acid synthesis substrate	FAO suppression; Lipogenesis
NADPH	Pentose phosphate pathway product	**↑**	NOX2-mediated ROS; Fatty acid synthesis	Pro-inflammatory (ROS source)
mtROS	ETC; reverse electron transport at SDH	**↑↑**	NLRP3 inflammasome activation; mtDNA damage; HIF-1α stabilization	Pro-inflammatory
Sadenosylmethionine	One-carbon metabolism	Altered	Histone methylation; Linked to glycolytic serine/glycine	Epigenetic
2-Oxoglutarate	TCA intermediate; PHD cofactor	**↓**	Required for PHD activity; Depletion favors HIF-1α stabilization	Indirectly pro-inflammatory

4-OI, 4-octyl itaconate; DI, dimethyl itaconate; IκBζ, inhibitor of kappa B zeta; Nrf2, nuclear factor erythroid 2-related factor 2; ROS, reactive oxygen species; mtROS, mitochondrial reactive oxygen species; ACC, acetyl-CoA carboxylase.

The symbol ↓ means "decrease", ↑ means "increase", ↑↑ means "significant increase", and → means "lead to" or "to".

- succinate accumulation upstream of succinate dehydrogenase (SDH) with consequent HIF-1α stabilization (Section 3.1.1). This mechanism is established in murine lipopolysaccharide-activated macrophages and bone marrow-derived macrophages (BMDMs) (11, 14); its direct demonstration in primary human hepatic macrophages from MASLD patients remains limited, though elevated hepatic succinate levels have been confirmed in human MASLD/MASH liver tissue by mass spectrometry imaging.
- In murine MASH models (HFD, CDAHFD) and ex vivo hMDMs treated with palmitic acid, β-arrestin 2 acts as a scaffold to recruit an as-yet-unidentified E3 ubiquitin ligase to immune-responsive gene 1 (IRG1, also known as aconitate decarboxylase 1 or ACOD1), promoting its proteasomal degradation and itaconate depletion; this relieves SDH inhibition and amplifies inflammatory signaling in macrophages (Section 3.1.3) (47, 48).
- Citrate export to the cytosol fuels fatty acid synthesis and protein acetylation, linking mitochondrial dysfunction to both lipotoxicity and epigenetic remodeling (Section 3.1.2) (49–52). This section examines each breakpoint in turn, tracing how these metabolic alterations create a self-amplifying inflammatory loop.

#### 3.1.1 TCA cycle disruption as a central hub: the succinate-HIF-1α axis

The TCA cycle, also known as the Krebs cycle or citric acid cycle, serves as the central hub of cellular metabolism, integrating the catabolism of carbohydrates, fats, and proteins while generating reducing equivalents for OXPHOS and biosynthetic precursors for anabolic processes (53). Evidence from inflammatory macrophage models, murine MASH models, and transcriptomic studies suggests that TCA-cycle remodeling may contribute to macrophage activation in MASLD; however, direct flux-based evidence in primary human hepatic macrophages remains limited. The cycle is rerouted at critical breakpoints that generate signaling metabolites with profound effects on inflammatory responses (54).

Under normal conditions, glucose-derived pyruvate enters the mitochondria and is converted to acetyl-CoA by the pyruvate dehydrogenase (PDH) complex. Acetyl-CoA then condenses with oxaloacetate to form citrate, initiating the TCA cycle (55). As citrate progresses through the cycle, it undergoes sequential oxidation reactions that generate reduced nicotinamide adenine dinucleotide (NADH) and FADH_2_, which fuel the electron transport chain (ETC), as well as GTP (or ATP) and CO_2_. The cycle is completed when oxaloacetate is regenerated, allowing continued acetyl-CoA oxidation.

In MASLD-associated macrophages, this orderly progression is disrupted at multiple points. The HIF-1α-mediated induction of pyruvate dehydrogenase kinase (PDK) enzymes inhibits PDH, reducing the entry of pyruvate-derived acetyl-CoA into the cycle (54, 56). However, the cycle can still be fueled by alternative substrates, particularly fatty acids, which are converted to acetyl-CoA through β-oxidation.

One candidate metabolic node implicated in inflammatory macrophage activation is the SDH-succinate axis, also known as mitochondrial complex II (14). SDH catalyzes the oxidation of succinate to fumarate, coupled with the reduction of flavin adenine dinucleotide (FAD) to FADH_2_. In inflammatory macrophages, succinate accumulates upstream of this reaction, creating a metabolic bottleneck (57). The accumulation of succinate has multiple functional consequences: it inhibits PHD enzymes, stabilizing HIF-1α and promoting glycolysis; it can be exported from mitochondria to function as a signaling metabolite through the succinate receptor SUCNR1; and it alters the redox state of the cell, affecting multiple metabolic and signaling pathways (58) (**Table 1**).

Succinate can inhibit 2-oxoglutarate (2-OG) -dependent dioxygenases, including PHDs, primarily by altering the intracellular succinate-to-2-OG ratio and through product inhibition, thereby favoring HIF-1α stabilization (11, 59–61). This mechanism was established in murine lipopolysaccharide-activated BMDMs, where succinate accumulation specifically drove HIF-1α-dependent IL-1β production (11), and elevated hepatic succinate has been confirmed in human MASLD liver tissue by mass spectrometry imaging (11, 14).

Under conditions permissive for reverse electron transport, succinate oxidation through SDH can contribute to mitochondrial ROS generation (14, 62). These ROS oxidize the PHD iron cofactor, indirectly stabilizing HIF-1α (63), and directly activate the NLRP3 inflammasome, linking succinate to IL-1β maturation through both transcriptional and post-translational mechanisms (62). Extracellular succinate signals through SUCNR1/GPR91, reinforcing HIF-1α-dependent programs and promoting macrophage recruitment (58, 64). In the steatotic liver, lipid overload, inflammatory cytokine signaling, and progressive hypoxia converge to sustain succinate accumulation, while itaconate depletion (Section 3.1.3) removes a critical brake on SDH activity, further amplifying the inflammatory signal (47, 48).

#### 3.1.2 Citrate accumulation and lipogenesis

Another critical alteration in the TCA cycle of MASLD-associated macrophages involves the accumulation and export of citrate. In the cytosol, citrate is cleaved by ACL to generate acetyl-CoA and oxaloacetate, providing a carbon source for fatty acid synthesis.

The increased availability of cytosolic acetyl-CoA in MASLD-associated macrophages drives multiple pathological processes. First, it serves as the substrate for fatty acid synthase, promoting the *de novo* synthesis of fatty acids that can be incorporated into lipid droplets or secreted as inflammatory mediators (65). Second, acetyl-CoA is the substrate for protein acetylation, linking citrate accumulation to epigenetic changes that promote inflammatory gene expression (66). Third, acetyl-CoA can be converted to malonyl-CoA by ACC, which not only provides another substrate for fatty acid synthesis but also allosterically inhibits CPT1, further suppressing FAO (67).

The citrate-ACL pathway has been identified as a critical node linking macrophage metabolism to inflammatory activation in both hepatocytes and macrophages (65, 68). Inhibition of ACL in macrophages reduces inflammatory cytokine production and improves insulin sensitivity in mouse models of obesity and MASLD (65). These findings highlight the therapeutic potential of targeting citrate metabolism in MASLD.

#### 3.1.3 Dynamic regulation of the IRG1-Itaconate axis: from early depletion to compensatory elevation

Itaconate is a TCA cycle-derived metabolite with potent anti-inflammatory properties that is produced by the enzyme IRG1 (69). IRG1 catalyzes the decarboxylation of cis-aconitate, a TCA cycle intermediate, to produce itaconate, which is then exported from mitochondria to the cytosol where it exerts its immunomodulatory effects (70). Under normal inflammatory conditions, itaconate production serves as a negative feedback mechanism that limits excessive activation.

In MASLD, the regulation of the IRG1-itaconate axis is complex and stage-dependent. During early-stage simple steatosis, resident Kupffer cells undergo apoptotic loss, leading to a relative depletion of itaconate-producing cells and reduced hepatic itaconate levels. However, as disease progresses to MASH, infiltrating monocyte-derived macrophages upregulate IRG1 expression in an Interferon α/β Receptor-dependent manner, driving marked itaconate accumulation in the liver (71). Importantly, β-arrestin 2-mediated IRG1 degradation operates as a counter-regulatory mechanism that restrains this compensatory itaconate response, as detailed below.

Wei et al. (47) identified a critical post-translational mechanism underlying itaconate depletion in murine MASLD-associated macrophages. In murine NASH models (CDAHFD, Western diet) and in hMDMs exposed to metabolic stress, hepatic macrophages exhibit elevated β-arrestin 2 expression, driven by sustained inflammatory signaling through the NF-κB pathway. This IRG1 destabilization acts as a counter-regulatory brake that limits the compensatory itaconate response observed in advanced MASH, thereby preventing full anti-inflammatory resolution and perpetuating inflammatory signaling.

Mechanistically, β-arrestin 2 serves as a scaffold to recruit the E3 ubiquitin ligase MDM2 to IRG1, promoting K48-linked polyubiquitination and subsequent proteasomal degradation of IRG1 (47). This modification targets IRG1 for rapid proteasomal degradation, with a half-life reduction from approximately 8 hours to under 2 hours in MASLD macrophages. Genetic deletion of β-arrestin 2 in myeloid cells (Arrb2^f/f^ Lyz2^Cre^ mice) restores IRG1 protein levels, increases itaconate production, and attenuates MASH progression even under sustained high-fat diet challenge. Conversely, myeloid-specific β-arrestin 2 overexpression exacerbates hepatic inflammation and fibrosis.

This β-arrestin 2-IRG1-itaconate axis represents a novel metabolic checkpoint that links inflammatory signaling to TCA cycle integrity. Therapeutically, strategies that disrupt the β-arrestin 2-IRG1 interaction, such as small molecule inhibitors or competing peptides derived from the IRG1 ubiquitination site, could potentially restore itaconate production and suppress hyperinflammation in MASLD.

Recent comprehensive reviews have further elaborated on the therapeutic potential of itaconate-based metabolic reprogramming, positioning the IRG1-itaconate axis as a tractable target for inflammatory diseases beyond MASLD (72).

The consequences of impaired itaconate signaling in MASLD-associated macrophages are significant. In the context of β-arrestin 2-mediated IRG1 degradation, itaconate-mediated inhibition of SDH is insufficient, leading to increased succinate oxidation and ROS production (73). Glycolysis proceeds unabated, fueled by HIF-1α stabilization and insufficiently restrained by itaconate-mediated suppression of glycolytic enzymes (74). Inflammatory signaling through the NF-κB pathway is amplified, as itaconate-mediated alkylation of IκBζ cysteine residues that would normally suppress interleukin-6 (IL-6) production is compromised (75). Notably, global or myeloid-specific Irg1 knockout mice exhibit exacerbated hepatic lipid accumulation, glucose intolerance, and insulin resistance under Western diet feeding, demonstrating that even the compensatory itaconate elevation observed in MASH is insufficient to fully counteract disease progression (71). Collectively, these effects create a hyperinflammatory state that drives MASLD progression.

The IRG1-itaconate axis exhibits marked stage-dependent dynamics during MASLD progression. Single-cell transcriptomic analysis reveals that IRG1 expression is detectable in resident Kupffer cells under steady-state conditions but declines dramatically with Western diet feeding, while robust IRG1 expression emerges in infiltrating Clec4f^–^ macrophages derived from circulating monocytes after 12–36 weeks of WD feeding (34). Mass spectrometry confirms that itaconate accumulates specifically in F4/80^+^ hepatic macrophages, with liver tissue concentrations reaching 400 ± 131 μM in WD-fed mice, whereas circulating plasma itaconate remains unchanged (71). In human NASH livers, IRG1 mRNA and itaconate levels are significantly elevated compared to non-NASH controls (109 ± 9 μM), whereas plasma itaconate shows no significant difference (71). These findings establish a clear stage-dependent trajectory: early depletion in simple steatosis (due to KC loss) followed by marked upregulation in MASH (driven by infiltrating macrophages), with β-arrestin 2 acting as a counter-regulatory brake that limits the full therapeutic potential of this compensatory response. Future studies combining cell-resolved metabolomics, stable-isotope tracing, and human liver tissue analysis are required to further define the temporal dynamics and functional relevance of endogenous itaconate across MASLD stages.

### 3.2 Metabolic collapse: from oxidative balance to glycolytic lock

**Figure 3:**
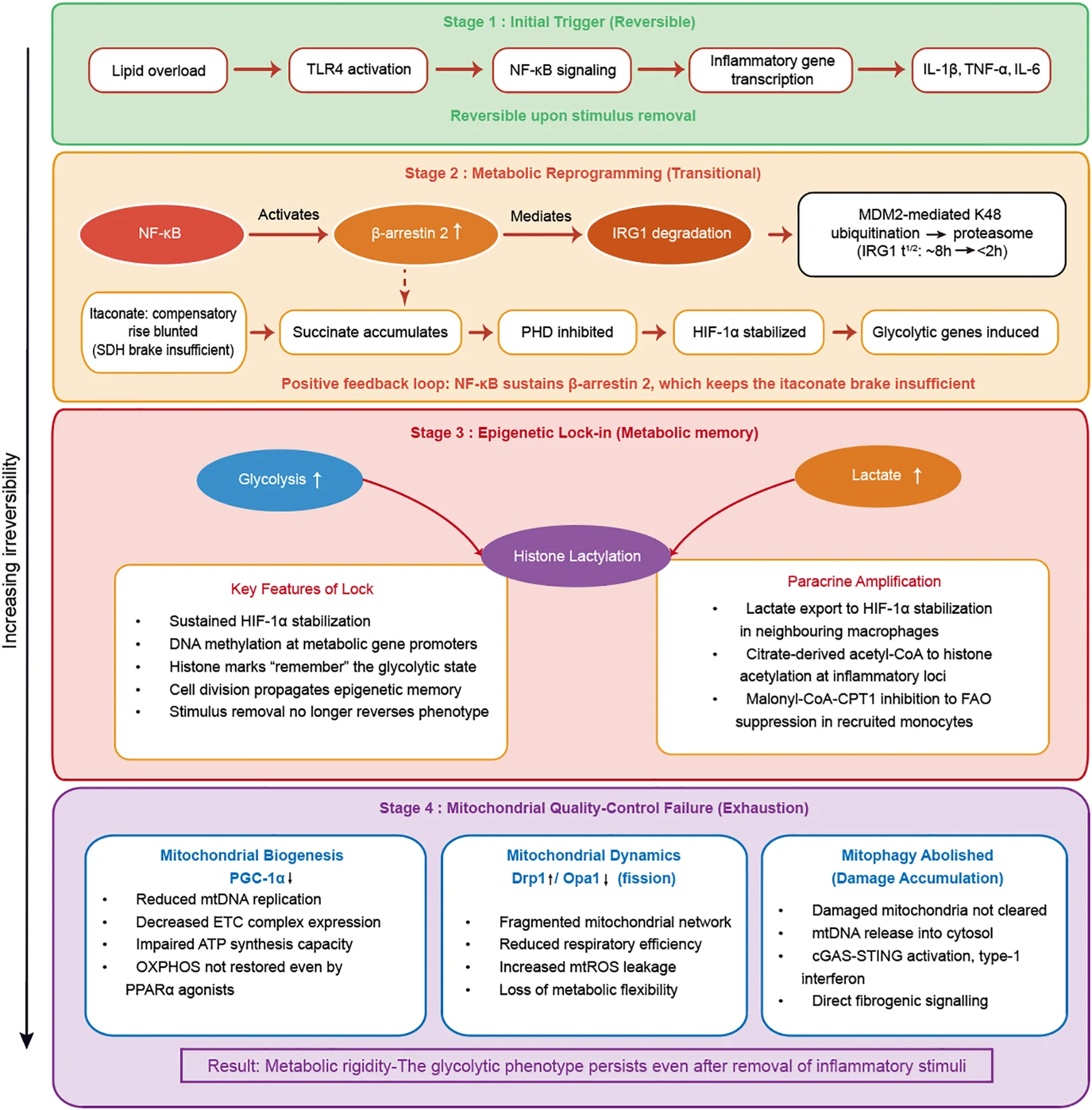
Metabolic-epigenetic coupling and glycolytic lock in MASLD macrophages: a four-stage progression from reversible inflammation to terminal metabolic exhaustion. Stage 1 (reversible): lipid overload activates Toll-like receptor 4 (TLR4)/NF-κB signaling, inducing IL-1β, TNF-α, and IL-6; reversible upon stimulus removal. Stage 2 (transitional): NF-κB upregulates β-arrestin 2, which scaffolds MDM2-mediated K48 ubiquitination and proteasomal degradation of IRG1 (half-life reduced from ~8 h → < 2 h); the compensatory itaconate rise is blunted, leaving the SDH brake insufficient, so succinate accumulates, PHD is inhibited, HIF-1α is stabilized, and glycolytic genes are induced. Stage 3 (epigenetic lock-in): persistent glycolysis and lactate accumulation drive histone lactylation (H3K18la), establishing metabolic memory (sustained HIF-1α, DNA methylation, epigenetic propagation through cell division) with paracrine amplification to neighboring macrophages. Stage 4 (exhaustion): mitochondrial quality control fails on three fronts-PGC-1α downregulation (biogenesis), Drp1 up/Opa1 down (dynamics), and abolished mitophagy with mtDNA-driven cGAS-STING activation and fibrogenic signaling. The result is metabolic rigidity: the glycolytic phenotype persists even after removal of inflammatory stimuli, driving irreversible MASH progression.

The transition from metabolic health to disease in MASLD involves divergent metabolic reprogramming across distinct hepatic macrophage populations. This chapter delineates the collapse of oxidative homeostasis and the establishment of a ‘glycolytic lock’, a state of metabolic inflexibility enforced by pseudohypoxia and mitochondrial dysfunction. We explore how this metabolic reprogramming extends beyond mere energy production to actively shape epigenetic landscapes and lipid handling, thereby locking macrophages into a persistent pro-inflammatory phenotype (**Figure 3**).

#### 3.2.1 HIF-1α stabilization and the pseudowarburg effect

A defining feature of hepatic macrophages in MASLD is the shift from oxidative metabolism to aerobic glycolysis, a metabolic reprogramming that occurs even in the presence of adequate oxygen, a phenomenon analogous to the Warburg effect described in cancer cells (76). This metabolic shift has been demonstrated in murine MASH liver non-parenchymal cells, *in vitro* polarized human macrophages (THP-1, hMDMs), and inferred from human MASLD liver transcriptomic data; direct metabolic flux analysis of primary human hepatic macrophages remains limited due to cell isolation challenges. This glycolytic commitment is enforced primarily through the stabilization and activation of HIF-1α, a master transcriptional regulator of cellular adaptation to hypoxia that also integrates signals from metabolic stress and inflammatory stimuli (77).

Under normoxic conditions, HIF-1α is continuously synthesized but rapidly degraded through a process mediated by PHD enzymes, which hydroxylate specific proline residues in the oxygen-dependent degradation domain of HIF-1α (59). This hydroxylation creates a recognition site for the von Hippel-Lindau E3 ubiquitin ligase, which targets HIF-1α for proteasomal degradation. The activity of PHD enzymes depends on molecular oxygen, iron, and 2-oxoglutarate, making HIF-1α degradation exquisitely sensitive to cellular oxygen tension (60).

In MASLD, HIF-1α stabilization occurs through multiple mechanisms. First, in human MASLD/MASH liver tissue and murine NASH models, the progressive fibrosis and architectural distortion of the liver parenchyma lead to genuine tissue hypoxia, particularly in the pericentral regions where oxygen extraction is highest (78). Second, the accumulation of ROS in steatotic hepatocytes and activated macrophages can oxidize the iron cofactor required for PHD activity, impairing HIF-1α hydroxylation (63). Third, and perhaps most importantly in the context of MASLD, the disruption of the TCA cycle leads to succinate accumulation, which stabilizes HIF-1α through the mechanisms detailed in Section 3.1.1 (pseudohypoxia via PHD inhibition, ROS-mediated PHD cofactor oxidation, and SUCNR1 signaling) (11, 58, 61). Notably, while itaconate normally inhibits SDH to limit succinate oxidation, the counter-regulatory β-arrestin 2-mediated IRG1 degradation in MASLD macrophages compromises this brake, allowing unchecked succinate accumulation and HIF-1α stabilization despite the compensatory itaconate elevation observed in advanced disease stages.

Once stabilized, HIF-1α forms a heterodimer with HIF-1β and binds to hypoxia-responsive elements in the promoters of target genes, initiating a transcriptional program that promotes glycolytic metabolism (79). HIF-1α upregulates the expression of glucose transporter (GLUT) 1/3, facilitating increased glucose uptake into macrophages. It also induces the expression of nearly all glycolytic enzymes, including hexokinase 2 (HK2), phosphofructokinase-1, and pyruvate kinase M2 (PKM2), thereby increasing glycolytic flux (80). Simultaneously, HIF-1α suppresses mitochondrial oxidative metabolism through multiple mechanisms, including the induction of PDK enzymes, which phosphorylate and inhibit the PDH complex, thereby blocking the entry of pyruvate into the TCA cycle (81).

The functional consequences of HIF-1α-driven glycolytic reprogramming in hepatic macrophages are profound. Glycolysis provides rapid, albeit less efficient, ATP generation that supports the energy demands of pro-inflammatory effector functions (9, 82). More importantly, glycolytic intermediates serve as biosynthetic precursors for the production of inflammatory mediators. For example, glucose-6-phosphate can be diverted into the pentose phosphate pathway to generate NADPH, which is required for NADPH oxidase-mediated ROS production and for fatty acid synthesis (83).

#### 3.2.2 Epigenetic remodeling by metabolic signals

Beyond its immediate effects on energy metabolism, the glycolytic shift in MASLD-associated macrophages has profound implications for epigenetic regulation. The metabolic state of a cell directly influences the availability of substrates for epigenetic modifying enzymes, thereby linking metabolic reprogramming to long-term changes in gene expression (49). This metabolic-epigenetic crosstalk represents a critical mechanism by which transient metabolic alterations can be “remembered” and perpetuated, contributing to the sustained inflammatory phenotype observed in chronic MASLD.

One of the most important metabolic-epigenetic connections involves histone acetylation. Acetyl-CoA, the substrate for histone acetyltransferases, is generated primarily from glucose-derived pyruvate through the action of the PDH complex and ACL (84). In glycolytic macrophages, increased glucose uptake and metabolism lead to elevated acetyl-CoA levels, which in turn promote histone acetylation at promoters of pro-inflammatory genes, enhancing their transcription (49).

Histone methylation is similarly influenced by cellular metabolism. S-adenosylmethionine, the methyl donor for histone methyltransferases, is generated through one-carbon metabolism, which is closely linked to glycolysis through the provision of serine and glycine (85).

Histone lactylation functions as a context-dependent metabolic-epigenetic switch transducing glycolytic flux into divergent transcriptional programs (86). Histone lactylation has been linked to elevated lactate and may involve enzymatic and non-enzymatic mechanisms; the precise endogenous acyl donor and the relative contribution of p300/CBP remain under investigation (86). In murine wound-healing models and *in vitro* polarized human macrophages, H3K18la activates wound-healing genes (Lrg1, Vegf-a, IL-10) (87). In murine NASH liver non-parenchymal cells and human MASLD liver tissue (immunohistochemistry and ChIP-seq data), it drives fibrogenic and inflammatory gene expression (CD86, iNOS) (86, 88). This functional duality depends on macrophage subset, disease stage, and temporal kinetics, lactylation’s slower dynamics (~24 h) enable a “metabolic memory” that can shift from inflammatory to resolution-phase programming (87). In MASLD, chronic hepatic lactate elevation likely biases this balance toward inflammatory signatures, though reparative lactylation may persist in specific microenvironments (**Table 2**).

**Table 2:** Metabolic comparison.

Metabolic feature	Healthy KCs	MASLD macrophages	Key molecules	References	Primary evidence
Primary energy metabolism	FAO and OXPHOS dominant	Aerobic glycolysis (Warburg-like)	FAO enzymes, ETC complexes	(9, 10, 59, 89)	KC oxidative profile: murine + human liver tissue; glycolytic shift: murine MASH non-parenchymal cells, human THP-1/hMDM *in vitro*, human transcriptomics
Glucose uptake	Low	High (GLUT1/3 ↑)	GLUT1, GLUT3, HK2, PFKFB3	(59, 82)	Murine models; human *in vitro* systems
TCA cycle flux	Complete oxidation	Rerouted; breakpoints at SDH, PDH	SDH, PDH, PDK	(11, 13, 56, 58, 80)	Murine BMDMs/MASH models; hepatic succinate confirmed in human MASLD by MS imaging
HIF-1α status	Low (PHD-active, von Hippel-Lindau-mediated degradation)	Stabilized (pseudohypoxia)	PHD2, von Hippel-Lindau, succinate	(11, 13, 61, 78–80)	Murine models; human MASLD/MASH tissue (hypoxia inferred)
PPARα/CPT1	High; drives FAO	Downregulated	PPARα, CPT1α, PGC-1α	(89–93)	Murine MASH (HFD, ob/ob); human transcriptomic inference
FAO	Robust	Impaired; paradoxical lipid accumulation	CPT1, ACOX1, MCAD	(9, 94–96)	Murine models; human *in vitro*
Mitochondrial function	Intact; efficient ATP synthesis	Dysfunctional; fragmented network	PGC-1α, Drp1, Opa1	(97–100)	Predominantly murine
ROS production	Low basal levels	mtROS ↑; mtDNA ↑; cardiolipin; NLRP3 activation	mtDNA, mtROS, cardiolipin, SOD2, MAVS/Mfn2	(62, 95, 101, 102)	Murine + *in vitro* human systems
Epigenetic marks	Baseline acetylation	Histone lactylation ↑; Acetylation ↑	p300/CBP, histone deacetylases, SIRT3	(68, 85, 86, 88, 103, 104)	Murine NASH NPCs; human MASLD liver (immunohistochemistry, ChIP-seq)
Cytokine profile	Anti-inflammatory: IL-10, TGF-β	Pro-inflammatory: IL-1β, TNF-α, IL-6	NF-κB, STAT1, IRF5	(105, 106)	Human + murine
Lipid handling	Scavenging; no accumulation	CD36-mediated uptake; lipotoxicity	CD36, FABP4, lipid droplets	(95, 96, 107)	Human + murine
Efferocytosis	Efficient clearance of apoptotic cells	Impaired (TREM2 shedding)	TREM2, DAP12, MerTK, Tim-4	(33, 34, 108, 109)	Murine MASH models; human NASH explants; THP-1 cells
Key macrophage populations	Resident KCs (80%-90%)	MoMFs recruited; TREM2^+^ LAMs	CCR2, CCL2, TREM2, CD9	(27, 33, 42)	Human scRNA-seq + murine models

CD36, cluster of differentiation 36; PPARα, peroxisome proliferator-activated receptor alpha.

The symbol ↑ means "increase".

#### 3.2.3 Impaired FAO and lipotoxicity

While MASLD-associated macrophages shift toward glycolytic metabolism, their capacity for FAO is markedly impaired. This decline in oxidative metabolism is not merely a consequence of substrate competition, where glucose metabolism suppresses lipid utilization, but rather reflects active transcriptional and post-translational downregulation of the FAO machinery (103). The coordinated suppression of PPARα and CPT1, two critical regulators of FAO, plays a central role in this metabolic reprogramming.

PPARα is a nuclear receptor that functions as a master transcriptional regulator of genes involved in fatty acid uptake, transport, and oxidation (104). In hepatocytes and macrophages, PPARα activation induces the expression of CPT1, the rate-limiting enzyme in mitochondrial fatty acid import, as well as multiple enzymes involved in β-oxidation and the TCA cycle (94). Under physiological conditions, PPARα activity is maintained by endogenous ligands such as fatty acids and their derivatives, ensuring robust FAO capacity in the liver (90).

In murine MASH models (HFD, ob/ob) and inferred from human MASLD liver transcriptomic data, hepatic macrophages exhibit marked downregulation of PPARα expression and activity, distinct from the hepatocyte-centric PPARα function in whole-body fatty acid homeostasis (91, 110). Additionally, HIF-1α, which is stabilized in MASLD-associated macrophages, can directly suppress PPARα transcription in certain cell types and interfere with its coactivator recruitment (111). The resulting decline in PPARα activity leads to reduced expression of CPT1 and other FAO enzymes, effectively crippling the ability of macrophages to oxidize fatty acids.

The inability of macrophages to perform FAO creates a paradoxical situation in MASLD. Despite being surrounded by an excess of lipids in the steatotic liver, macrophages cannot effectively utilize these fatty acids as an energy source. Instead, they continue to take up lipids through scavenger receptors such as CD36, leading to intracellular lipid accumulation and the formation of lipid droplets (92). This ectopic lipid storage triggers lipotoxicity, characterized by ER stress, mitochondrial dysfunction, and the activation of inflammatory signaling pathways including the NLRP3 inflammasome (16).

Lipotoxicity in macrophages contributes to disease progression in multiple ways. Saturated fatty acids can potentiate TLR4-associated inflammatory signaling, although whether they act as direct TLR4 ligands remains debated (93). Lipid-induced ER stress activates the unfolded protein response, which can promote inflammation through multiple mechanisms including the activation of JNK and NF-κB signaling (96). Furthermore, lipid overload in mitochondria impairs their function, leading to increased ROS production and further metabolic disruption (112).

#### 3.2.4 Mitochondrial dysfunction and quality control

The metabolic reprogramming of hepatic macrophages in MASLD is accompanied by profound mitochondrial dysfunction that extends beyond passive metabolic adaptation to actively drive inflammatory signaling. This section integrates three interconnected mechanisms: intrinsic mitochondrial quality control failure, TREM2-dependent metabolic regulation, and the metabolic consequences of efferocytosis impairment (phenotypic aspects discussed in Section 2.3).

(1) Mitochondrial quality control failure.

HIF-1α-mediated induction of PDK enzymes inhibits the PDH complex, reducing pyruvate entry into the TCA cycle and limiting NADH and FADH_2_ generation for the ETC (113). Additionally, the accumulation of damaged mitochondria and the impaired clearance of these organelles through mitophagy contribute to ETC dysfunction (107). The resulting decrease in mitochondrial membrane potential and ATP synthesis capacity further reinforces glycolytic commitment. This ETC disruption is compounded by impaired mitochondrial quality control programs. PGC-1α, the master regulator of mitochondrial biogenesis, is downregulated in MASLD-associated macrophages (101), while mitochondrial dynamics shift toward excessive fission, fragmenting the network (102).

(2) TREM2 as a Metabolic Regulator.

The ADAM17-mediated TREM2 shedding described in Section 2.3.3 has profound metabolic consequences beyond efferocytosis failure. TREM2-DAP12 signaling normally activates the mTORC2-IRF4 axis to promote oxidative metabolism and suppress glycolysis; TREM2 loss therefore removes a critical metabolic brake, accelerating glycolytic commitment (108). Furthermore, uncleared apoptotic hepatocytes from impaired efferocytosis release saturated fatty acids that directly suppress mitochondrial FAO in both LAMs and newly recruited MoMFs (Section 3.2.3), reinforcing the metabolic-inflammatory vicious cycle.

The concept of hepatic mitochondria as a systemic signaling hub extends beyond local MASLD pathogenesis. Mitochondrial stress in hepatic macrophages generates circulating signals, including unfolded protein response mitochondrial-driven mitokines, namely fibroblast growth factor 21 (FGF21) and growth differentiation factor 15, TCA cycle metabolites, and mitochondrial danger signals (mtROS, mtDNA), that reshape inflammation and energy homeostasis across distant organs, positioning MASLD-associated macrophage mitochondrial dysfunction within a broader inter-organ communication network (114).

### 3.3 Gut-liver axis and microbiome-derived metabolites in macrophage immunometabolism

The gut-liver axis constitutes a bidirectional communication system wherein microbiome-derived metabolites serve as critical signaling molecules modulating hepatic macrophage immunometabolism, a dimension that warrants explicit discussion in the context of MASLD pathogenesis. Short-chain fatty acids (SCFAs), primarily acetate, propionate, and butyrate, produced by bacterial fermentation of dietary fibers, exert multifaceted immunometabolic effects on macrophage polarization and function. Butyrate, in particular, inhibits histone deacetylases in macrophages, thereby suppressing pro-inflammatory NF-κB signaling and promoting an anti-inflammatory phenotype, while concurrently enhancing intestinal barrier integrity via upregulation of tight junction proteins such as claudin-1 and zonula occludens-1, thereby attenuating endotoxin-mediated hepatic inflammation (115, 116). Furthermore, SCFAs activate G-protein-coupled receptors (GPR41/FFAR3 and GPR43/FFAR2) on macrophages, triggering downstream calcium/calmodulin-dependent protein kinase II and cAMP response element-binding protein signaling, which orchestrates metabolic reprogramming toward FAO and away from *de novo* lipogenesis (117).

Bile acids (BAs), another class of pivotal microbiome-modulated metabolites, function as endocrine signaling molecules that profoundly shape macrophage immunometabolism through the farnesoid X receptor (FXR) and Takeda G protein-coupled receptor 5 (TGR5). The gut microbiota-mediated biotransformation of primary to secondary BAs alters the BA pool composition, which is markedly disrupted in MASLD and MASH. TGR5 activation on KCs and recruited macrophages can polarize cells toward an anti-inflammatory alternatively activated macrophage (M2)-like phenotype; conversely, TGR5 deficiency promotes NLRP3 inflammasome activation and caspase-1 cleavage, driving classically activated polarization and exacerbating liver injury and inflammation (118). Additionally, FXR signaling in monocytes and macrophages suppresses NF-κB-mediated inflammatory responses, positioning BA receptor modulation as an immunometabolic therapeutic strategy (119).

Collectively, these microbiome-derived metabolites illustrate how intestinal dysbiosis propagates hepatic immunometabolic dysfunction through the gut-liver axis. The reduction in SCFA-producing taxa (e.g., Faecalibacterium, Roseburia) and the concomitant alteration in BA profiles observed in MASLD patients directly impinge upon macrophage metabolic programming, linking microbial ecology to innate immune effector function. Integrating these gut-derived signals into the immunometabolic framework of MASLD not only addresses the mechanistic gap identified but also underscores the therapeutic potential of microbiome-targeted interventions, such as precision prebiotics, engineered probiotics, or fecal microbiota transplantation, to restore immunometabolic homeostasis in hepatic macrophages.

Recent work has framed MASH progression as a gradual loss of immunometabolic adaptability along the gut-liver axis, wherein reductions in SCFAs and secondary BAs, together with increased succinate and endotoxin levels, alter KC and infiltrating macrophage metabolism through FXR/TGR5 and mTOR/adenosine monophosphate-activated protein kinase signaling, favoring glycolytic pro-inflammatory phenotypes associated with HIF-1α stabilization and NLRP3 inflammasome activation (64).

## 4 Metabolic reprogramming across MASLD stages

**Figure 4:**
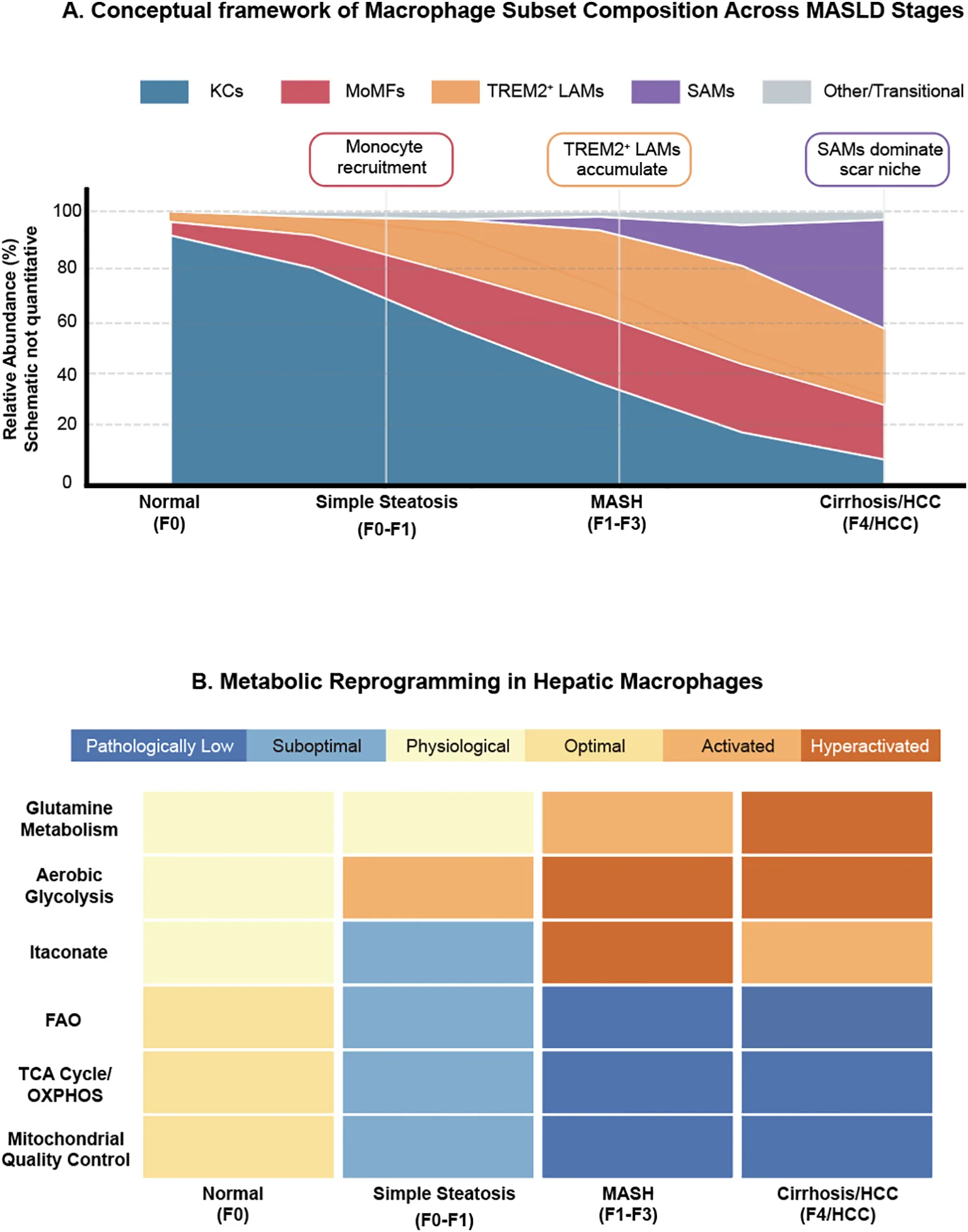
Dynamic remodeling of hepatic macrophage landscape during MASLD progression. **(A)** Schematic stacked area chart depicting conceptual shifts in the relative abundance (%) of hepatic macrophage subsets across MASLD stages: normal liver (F0), simple steatosis (F0-F1), MASH (F1-F3), and cirrhosis/HCC (F4/HCC). Proportional relationships are illustrative rather than quantitative, as absolute values vary substantially across species (mouse vs human) and analytical platforms (scRNA-seq vs flow cytometry). **(B)** Metabolic reprogramming in hepatic macrophages. Heatmap showing divergent metabolic trajectories across liver disease stages (Normal, steatosis, MASH, and cirrhosis/HCC). Processes are grouped as upregulated (glutamine metabolism, and aerobic glycolysis) or downregulated (FAO, TCA cycle/OXPHOS, and mitochondrial quality control) with disease progression; itaconate exhibits a U-shaped trajectory: downregulated in steatosis, upregulated in MASH and cirrhosis. Colors denote functional status on a diverging scale (blue: suboptimal/pathologically low; yellow: physiological/optimal; orange-red: activated/hyperactivated).

The pathological progression of MASLD unfolds through distinct stages, from simple steatosis, through MASH, to fibrosis and ultimately cirrhosis, during which the hepatic macrophage pool undergoes substantial compositional and metabolic remodeling with marked temporal and spatial heterogeneity across subpopulations (**Figure 4**). This section systematically dissects stage-specific metabolic characteristics and functional consequences of individual macrophage subsets.

### 4.1 Simple steatosis: subclinical oxidative metabolic stress

During early MASLD, in murine models (short-term HFD vs chow diet controls), the hepatic macrophage pool remains dominated by resident KCs (> 80% in rodents), with minimal MoMF infiltration and absence of LAM or SAM populations (18). At this stage, metabolic alterations are subtle and largely compensatory.

Metabolic phenotype: TCA cycle flux decreases globally but without discrete nodal breakpoints; succinate levels remain below the threshold for stable HIF-1α stabilization. Itaconate levels decrease during this stage due to apoptotic loss of IRG1-expressing resident Kupffer cells, creating a relative deficiency in this anti-inflammatory metabolite.

Functional consequences: Despite these subclinical metabolic shifts, KC tolerogenic functions remain largely preserved. The liver maintains immune homeostasis, and inflammation is minimal. However, the early reduction in itaconate and modest mitochondrial stress represent pre-pathological changes that prime the system for subsequent metabolic collapse upon sustained metabolic challenge (35, 47, 48, 120). Notably, this early itaconate depletion precedes the compensatory upregulation that characterizes advanced MASH stages.

Transition risk: The critical question at this stage is what determines progression versus regression. Emerging evidence suggests that the rate of KC turnover and the efficiency of mitochondrial quality control are key variables: impaired mitophagy in KCs accelerates their apoptotic loss, creating the niche for MoMF recruitment that defines the transition to MASH (30).

### 4.2 MASH phase: establishment of glycolytic commitment and subset divergence

Upon progression to MASH, in murine MASH models (CDAHFD, AMLN diet, Western diet) as confirmed in human MASH liver tissue by single-cell RNA-seq and immunohistochemistry, the hepatic macrophage compartment undergoes profound compositional remodeling: MoMFs surge into the liver and may constitute 30%-50% of total hepatic macrophages, while TREM2-expressing LAMs expand markedly, assembling into characteristic crown-like structures (18, 121). KC proportion decreases relatively, with increased phenotypic heterogeneity (122).

Spatial metabolic gradient: Pericentral macrophages exhibit more pronounced glycolytic commitment due to local hypoxia (lower PO_2_), whereas periportal populations retain relatively greater oxidative capacity (66). This zonation creates metabolic micro-niches that shape macrophage functional diversity.

The metabolic lock-in point: A pivotal event in MASH progression is the establishment of self-reinforcing metabolic-epigenetic coupling. Persistent lactate accumulation and histone lactylation have been proposed as potential mechanisms linking glycolytic reprogramming to sustained transcriptional changes; however, the existence of a durable epigenetic memory in hepatic macrophages during human MASLD remains to be established (123). This “metabolic memory” transforms transient metabolic adaptation into stable inflammatory phenotype propagation.

Itaconate dynamics: A hallmark of the MASH phase is the marked upregulation of the IRG1-itaconate axis. Infiltrating monocyte-derived macrophages (Clec4f^–^ population) upregulate IRG1 expression in an Interferon α/β Receptor-dependent, Stat1-independent manner, driving hepatic itaconate accumulation to 400 ± 131 μM in murine models and 109 ± 9 μM in human NASH livers (71). This represents a compensatory anti-inflammatory response; however, concurrent β-arrestin 2-mediated IRG1 degradation acts as a counter-regulatory brake, limiting the full therapeutic potential of this endogenous itaconate surge. Myeloid-specific β-arrestin 2 knockout restores IRG1 protein levels and itaconate production, attenuating MASH progression (47), confirming that the failure lies not in itaconate production per se, but in the insufficient magnitude of the compensatory response relative to inflammatory drive.

TREM2 checkpoint: LAMs at this stage retain membrane-bound TREM2 and functional efferocytosis, but ADAM17 activity is heightened, positioning them at a critical phenotypic inflection point (102).

### 4.3 Fibrotic stage: collapse of mitochondrial quality control and failure of repair programs

The defining feature of fibrotic MASLD is the emergence and expansion of SAMs within fibrous septa, alongside the metabolic convergence of previously distinct macrophage subsets toward a unified dysfunctional state (124).

SAM metabolic programming: SAMs depend on the synergistic interplay between glycolysis and glutaminolysis to provide precursors for collagen synthesis (125). Their metabolic profile is distinct from both MoMFs and LAMs: they exhibit sustained glycolytic flux coupled with active glutamine uptake, supporting fibrogenic effector functions.

LAM metabolic collapse: TREM2^+^ LAMs undergo a critical transition from “lipid-overload metabolism” to “metabolic exhaustion”. ADAM17-mediated TREM2 cleavage (Section 2.3.3) triggers this collapse, impairing efferocytosis and leading to apoptotic cell secondary necrosis (126). Crucially, their metabolic state converges with that of pro-inflammatory MoMFs: both subsets now exhibit glycolytic lock, creating a unified “lipid-metabolism-inflammation” crisis that characterizes advanced MASH. Despite the compensatory itaconate elevation observed in MASH, the metabolic exhaustion of LAMs and the counter-regulatory β-arrestin 2-mediated IRG1 degradation prevent full anti-inflammatory resolution, leaving the glycolytic-inflammatory program unchecked.

KC terminal state: Residual KCs exhibit diminished metabolic plasticity. Persistent epigenetic modifications (histone lactylation, DNA methylation) “lock in” the glycolytic program, while compromised mitochondrial quality control and abolished mitophagic capacity result in metabolic rigidity (127). These cells can no longer revert to oxidative metabolism even upon removal of inflammatory stimuli.

Mitochondrial danger signaling: Dysfunctional mitochondria accumulate due to impaired mitophagy, releasing mtDNA that activates the cGAS-stimulator of interferon genes pathway, directly linking metabolic failure to fibrogenic signaling (127).

Irreversibility threshold: The fibrotic stage likely represents a point of limited metabolic plasticity. Epigenetic modifications become entrenched, and structural changes in the microenvironment create physical barriers to metabolic normalization. This has critical implications for therapeutic timing (Section 5.1).

### 4.4 Cirrhosis and HCC: metabolic extremes and microenvironmental competition

At this advanced stage, traditional macrophage subset boundaries blur, and metabolic interactions with the tumor microenvironment dominate disease biology (128). Metabolic competition: Tumor cells engage in “metabolic hijacking”, consuming glucose and glutamine at high rates and compelling macrophages to shift toward alternative pathways. Macrophages in this context polarize toward a protumoral, immunosuppressive phenotype driven by metabolic competition rather than classical inflammatory cues (129).

Two metabolic extremes coexist: (i) metabolically exhausted macrophages, with compromised mitochondrial function and declining glycolytic efficiency, characterized by low ATP production and impaired phagocytic capacity; and (ii) lipid-overloaded, ferroptosis-sensitive macrophages, with heightened lipid accumulation and iron dysregulation create susceptibility to ferroptotic cell death, releasing pro-inflammatory lipid peroxides (130).

Therapeutic implications: The metabolic complexity of cirrhotic/HCC liver, characterized by hypoxia, acidosis, and nutrient competition, creates substantial barriers to metabolic therapy. Strategies effective in early MASLD may fail at this stage due to both metabolic heterogeneity and physical barriers imposed by fibrotic architecture.

Furthermore, vitamin D metabolism has emerged as a modifiable axis linking MASLD-associated metabolic stress to HCC progression, with implications for macrophage-mediated anti-tumor immunity (131).

## 5 Therapeutic leverage: metabolic plasticity and strategic exploration

The recognition that macrophage metabolic states are not fixed but plastic offers a transformative opportunity for therapeutic intervention. This chapter evaluates the feasibility of reversing the glycolytic lock by targeting key nodal points of immunometabolism, including nuclear receptors, glycolytic enzymes, and mitochondrial quality control pathways. We synthesize evidence from preclinical and clinical studies to propose that combinatorial strategies, informed by single-cell metabolic profiling, are essential to restore macrophage homeostasis and halt disease progression.

### 5.1 The principle of metabolic plasticity

A fundamental question in the development of metabolic therapies for MASLD is whether the metabolic states of hepatic macrophages are fixed or reversible. If the glycolytic, pro-inflammatory phenotype represents a terminal differentiation state, then therapeutic strategies would need to focus on removing or replacing these cells. However, if metabolic states exhibit plasticity, with the potential to revert to oxidative, anti-inflammatory profiles, then metabolic reprogramming becomes a viable therapeutic goal (132).

Accumulating evidence supports the concept of metabolic plasticity in macrophages. Early studies demonstrated that classically activated macrophage, glycolytic macrophages could be repolarized toward an M2-like, oxidative phenotype by treatment with IL-4 or other anti-inflammatory stimuli (133). This repolarization was accompanied by a metabolic shift from glycolysis to OXPHOS, indicating that the metabolic state is not fixed but can be modulated by environmental signals. More recent studies have shown that pharmacological interventions targeting specific metabolic enzymes can drive macrophage metabolic reprogramming and alter their functional phenotype (68).

The plasticity of macrophage metabolism is rooted in the interconnected nature of metabolic pathways. Glycolysis and oxidative metabolism are not isolated processes but are linked through multiple shared intermediates and regulatory mechanisms (134). Changes in one pathway can have ripple effects throughout the metabolic network, potentially shifting the balance between glycolytic and oxidative phenotypes. For example, enhancing FAO can reduce glycolytic flux by competing for cellular energy demand and by generating metabolites that inhibit glycolytic enzymes (135).

However, the extent of metabolic plasticity may be limited in chronic MASLD. Long-term exposure to inflammatory and metabolic stress can lead to epigenetic modifications that stabilize the glycolytic phenotype, making it more resistant to reversal (136). Additionally, the structural changes in the fibrotic liver microenvironment may create physical barriers that prevent the restoration of normal metabolic conditions (137). These considerations suggest that metabolic therapies may be most effective when applied early in disease progression, before irreversible epigenetic and structural changes have occurred.

The therapeutic targeting of hepatic macrophages in liver fibrosis has been systematically explored through multiple mechanistic avenues, including metabolic reprogramming, chemokine axis inhibition, and functional polarization modulation, underscoring the translational viability of macrophage-centered strategies (138).

### 5.2 PPAR agonists: restoring FAO

PPARs are nuclear receptors that function as master regulators of lipid metabolism and inflammation. Three isoforms exist, PPARα, PPARδ/β, and PPARγ, each with distinct tissue distributions and functional roles (139). In the context of MASLD, PPARα has been the primary target for therapeutic development due to its central role in regulating FAO and its expression in hepatocytes and macrophages (140).

PPARα agonists, including fibrates and more recently developed compounds such as elafibranor (PPARα/δ; GOLDEN-505 trial) (141) and the pan-PPAR agonist lanifibranor (NATIVE trial) (142), activate PPARα, inducing the expression of genes involved in fatty acid uptake, transport, and oxidation, including CPT1 and multiple β-oxidation enzymes (143). In macrophages, metabolic reprogramming toward OXPHOS and FAO attenuates inflammatory cytokine production (110). While PPARγ activation promotes alternative macrophage activation (144), PPARα primarily restores mitochondrial oxidative metabolism in the context of MASLD.

Elafibranor (GFT505), a dual agonist of PPARα and PPARδ, has emerged as a promising therapeutic candidate for MASLD, with clinical efficacy demonstrated in human Phase 2b trials (GOLDEN-505) and mechanistic insights derived from murine NASH models and human hepatocyte/hepatic stellate cell cultures (145). In the Phase 2b GOLDEN-505 trial, elafibranor at 120 mg achieved resolution of NASH without worsening of fibrosis in patients with active disease (NAS ≥ 4), alongside improvements in cardiometabolic risk profiles including reduced plasma triglycerides and low-density lipoprotein cholesterol, increased high-density lipoprotein cholesterol, and amelioration of insulin resistance and hepatic inflammatory markers (141). The mechanism of action involves cell type-specific dual receptor activation: in hepatocytes, PPARα enhances mitochondrial and peroxisomal FAO and lipolysis, thereby reducing steatosis and lipotoxicity (146); in hepatic macrophages (KCs), as demonstrated in murine liver non-parenchymal cells and hMDMs *in vitro*, PPARα and PPARδ cooperate to suppress TLR4/NF-κB-driven pro-inflammatory cytokine production and promote alternative M2 polarization, thereby limiting inflammation and fostering an anti-inflammatory hepatic microenvironment (146).

Lanifibranor, a pan-PPAR agonist that activates all three PPAR isoforms, has also shown promising results in clinical trials (142). In the NATIVE Phase 2b trial, lanifibranor treatment significantly improved the primary endpoint of decrease in the steatosis, activity, fibrosis-activity score and showed benefits on multiple histological parameters including fibrosis improvement (142). The pan-PPAR activity of lanifibranor may provide broader metabolic benefits compared to selective PPARα agonists, including improved insulin sensitivity through PPARγ activation and enhanced mitochondrial function through PPARδ activation (90).

### 5.3 Direct glycolytic inhibitors

Given the central role of glycolysis in maintaining the pro-inflammatory phenotype of MASLD-associated macrophages, direct inhibition of glycolytic enzymes represents an attractive therapeutic strategy. Multiple enzymes in the glycolytic pathway have been targeted, with varying degrees of success in preclinical models (147).

HK2, which catalyzes the first committed step of glycolysis by phosphorylating glucose to glucose-6-phosphate, has been a major target for glycolytic inhibition (148). HK2 is upregulated in inflammatory macrophages and is required for their glycolytic commitment and pro-inflammatory function (149). *In vitro* and in tumor models, HK2 inhibitors such as 2-DG and lonidamine can reduce glycolytic flux.

PKM2, which catalyzes the final step of glycolysis converting phosphoenolpyruvate to pyruvate, has also been targeted (150). PKM2 exists in multiple oligomeric states with different enzymatic properties, and the balance between these states is regulated by metabolic and signaling inputs. Small molecules that activate PKM2, promoting its tetrameric form with high pyruvate kinase activity, have been shown to suppress inflammatory macrophage activation and improve insulin sensitivity (151).

Lactate dehydrogenase A, which catalyzes the conversion of pyruvate to lactate, is another potential target (152). Inhibition of lactate dehydrogenase A not only reduces lactate production but also alters the cellular redox state by decreasing NADH consumption, potentially affecting multiple metabolic and signaling pathways (153). Lactate dehydrogenase A inhibitors have shown anti-inflammatory effects in various disease models, although their efficacy in MASLD specifically has not been extensively evaluated (154).

Despite promising preclinical results, the clinical development of direct glycolytic inhibitors faces significant challenges. Glycolysis is essential for the function of many cell types (147), and the redundancy of metabolic pathways means that cells can often compensate for the inhibition of a single enzyme (8). Strategies that achieve liver-specific or macrophage-specific glycolytic inhibition, perhaps through targeted drug delivery or the development of tissue-selective inhibitors, may be necessary to overcome these limitations.

### 5.4 Itaconate analogs and metabolic-immunomodulation

The recognition of itaconate as a critical anti-inflammatory metabolite has spurred interest in developing itaconate-based therapies for inflammatory diseases including MASLD (72, 75). Among these, 4-OI and DI are cell-permeable electrophilic itaconate derivatives widely used as pharmacological probes. Their intracellular metabolism and target engagement differ from those of endogenous itaconate; therefore, findings obtained with these derivatives should not be interpreted as direct evidence of endogenous itaconate function.

Both 4-OI and DI have shown potent anti-inflammatory effects in multiple preclinical models. These compounds inhibit the production of pro-inflammatory cytokines including IL-6, IL-1β, and TNF-α in macrophages stimulated with lipopolysaccharide or other inflammatory stimuli (155). The mechanisms underlying these effects include the inhibition of SDH, which reduces ROS production and HIF-1α stabilization; the alkylation of cysteine residues on kelch-like ech-associated protein 1, which activates the Nrf2 antioxidant pathway; and the direct modification of inflammatory signaling proteins including IκBζ (74).

In the context of MASLD, the therapeutic rationale for itaconate analogs is strongly supported by the observation that endogenous itaconate levels are already elevated in MASH (109-400 μM in liver tissue) yet remain insufficient to fully suppress inflammation, due in part to counter-regulatory β-arrestin 2-mediated IRG1 degradation (47). Exogenous administration of 4-OI (50 mg/kg in 40% cyclodextrin/phosphate-buffered saline for 12 weeks) reverses dyslipidemia and reduces hepatic lipid accumulation in Western diet-fed Irg1^^–^/^–^^ mice, with liver itaconate levels peaking at 4 hours post-administration and returning to baseline within 24 hours (71). Mechanistically, 4-OI reduces Oil Red O staining and mesenteric fat weight, and improves glucose tolerance in MASLD models. These findings establish that augmenting the endogenous compensatory itaconate response, overcoming the β-arrestin 2-mediated brake, represents a viable therapeutic strategy for MASLD. The dual metabolic and immunomodulatory effects of itaconate analogs make them particularly attractive candidates for MASLD therapy, as they can simultaneously address both the metabolic and inflammatory aspects of the disease.

Additionally, the optimal dosing, formulation, and route of administration for itaconate analogs have not been determined. Further preclinical and early clinical studies will be needed to evaluate the therapeutic potential of this approach (72).

### 5.5 CCR2/CCR5 inhibition: lessons from cenicriviroc

The recruitment of monocyte-derived macrophages from the circulation is a critical process in MASLD pathogenesis, driven primarily by the CCL2-CCR2 chemokine axis (156). Inhibition of this axis has been pursued as a therapeutic strategy, with the dual CCR2/CCR5 inhibitor cenicriviroc being the most extensively studied compound in this class (157).

Cenicriviroc is a dual inhibitor of CCR2 and CCR5, two chemokine receptors that mediate monocyte recruitment and macrophage activation (158). In preclinical models, cenicriviroc treatment reduces hepatic macrophage infiltration, improves insulin sensitivity, and attenuates fibrosis (158). These promising results led to the initiation of the Phase 3 AURORA trial, which evaluated cenicriviroc in patients with MASH and fibrosis (157). The AURORA trial enrolled 1,778 patients with NASH and stage 2/3 fibrosis, randomized to receive cenicriviroc 150 mg daily or placebo for 12 months (Part 1) with an optional extension to 60 months (Part 2). The primary endpoint, fibrosis improvement of ≥ 1 stage without worsening of steatohepatitis, was achieved in 22.3% of patients receiving cenicriviroc vs 25.5% in the placebo group (OR 0.84, 95% CI 0.63-1.10; P = 0.21). Similarly, complete resolution of steatohepatitis without fibrosis worsening occurred in 23.0% vs 27.2% of patients (P = 0.21) (159).

This disappointing outcome has provided important lessons for the development of macrophage-targeted therapies in MASLD. The failure of cenicriviroc suggests that simply blocking the recruitment of new macrophages may be insufficient to reverse established disease, particularly when resident macrophages and already-infiltrated monocyte-derived cells continue to drive inflammation and tissue damage (109).

The cenicriviroc experience highlights the need for combination approaches that address both the recruitment of new macrophages and the metabolic and functional state of existing macrophages in the liver (160). Strategies that combine CCR2/CCR5 inhibition with metabolic modulators, such as PPAR agonists or glycolytic inhibitors, could potentially achieve superior therapeutic outcomes by simultaneously reducing macrophage numbers and reprogramming the metabolism of residual cells (5).

The experience with cenicriviroc and other chemokine axis inhibitors illustrates both the promise and limitations of monocyte recruitment blockade as an anti-fibrotic strategy, emphasizing that combination approaches addressing both macrophage recruitment and metabolic reprogramming may be necessary (138).

### 5.6 FDA accelerated approval pharmacotherapies (2024-2026)

The period 2024–2026 has witnessed transformative developments in MASLD/MASH pharmacotherapy, with the first two FDA accelerated approval agents becoming available. These approvals represent a paradigm shift from lifestyle intervention as the sole therapeutic option to evidence-based pharmacological management of non-cirrhotic MASH with moderate-to-advanced fibrosis.

#### 5.6.1 Resmetirom (Rezdiffra™): first FDA accelerated approval MASH therapy

Resmetirom, a liver-selective thyroid hormone receptor-β agonist, received FDA accelerated approval in March 2024 as the first pharmacotherapy for non-cirrhotic MASH with moderate-to-advanced fibrosis (F2-F3). The pivotal MAESTRO-NASH Phase 3 trial (n=1,759) demonstrated that both 80 mg and 100 mg doses achieved the dual primary endpoints at 52 weeks: (i) MASH resolution (including reduction in NAS ≥ 2 points) without worsening of fibrosis, achieved in 30% (100 mg) vs 10% placebo; and (ii) fibrosis improvement by at least one stage without worsening of MASH, achieved in 26% vs 14% placebo (161). Resmetirom primarily acts by directly replenishing hepatic thyroid hormone receptor-β signaling, thereby promoting mitochondrial β-oxidation and fatty acid catabolism.

Macrophage-mediated considerations: Resmetirom’s primary mechanism is hepatocyte-directed metabolic improvement. However, the reduction in hepatocyte steatosis and lipotoxicity indirectly attenuates the pro-inflammatory milieu that drives macrophage activation. Decreased release of DAMPs and reduced hepatocyte apoptosis diminish KC activation and inflammatory monocyte recruitment. While direct macrophage-mediated effects of resmetirom are less well characterized compared to other drug classes, the histological resolution of inflammation observed in MAESTRO-NASH likely reflects, in part, this indirect macrophage modulation.

#### 5.6.2 Semaglutide (Wegovy^®^): second FDA accelerated approval MASH therapy

On August 15, 2025, the FDA granted accelerated approval to semaglutide 2.4 mg as the second MASH therapeutic for adults with moderate-to-advanced fibrosis (F2-F3), based on the Phase 3 ESSENCE trial results. The trial (n=800 in Part 1 interim analysis) demonstrated that 62.9% of patients achieved MASH resolution without fibrosis worsening vs 34.3% on placebo (estimated difference in percentage points 28.7%, 95% CI 21.1-36.2; P < 0.001), and 36.8% achieved fibrosis improvement without MASH worsening vs 22.4% on placebo (estimated difference in percentage points 14.4%, 95% CI 7.5-21.3; P < 0.001) at 72 weeks. A confirmatory secondary endpoint showed 32.7% achieved both endpoints vs 16.1% on placebo.

Critical mechanistic insight: *Post hoc* analyses presented at UEG Week 2025 and ECO 2025 revealed that 52%-72% of semaglutide’s effects on MASH-related endpoints and 48.9%-58.5% of fibrosis-related benefits were independent of weight loss. For MASH resolution, the odds ratio for weight loss-independent effect was 2.0 (1.4-3.0), representing 51.9% of the total benefit not mediated by weight reduction alone. For fibrosis improvement, the weight loss-independent OR was 1.5 (1.0-2.4), representing 55.5% of total benefit. These data confirm that semaglutide acts through multiple metabolic pathways extending beyond its well-known effects on body weight.

Direct macrophage-mediated mechanisms: Most of the clinical benefit of GLP-1 receptor agonists in MASH is likely mediated through systemic weight loss, improved insulin sensitivity, and reduced hepatocellular lipotoxicity. Direct effects on macrophages have been reported in preclinical systems but remain incompletely validated in human liver macrophages. Preclinical studies in murine macrophage cell lines (RAW264.7), BMDMs, and hMDMs indicate that GLP-1 receptor agonists can: (i) suppress pro-inflammatory cytokine production (TNF-α, IL-6, IL-1β) from macrophages; (ii) promote M2-like polarization through cyclic adenosine monophosphate/protein kinase A signaling; (iii) reduce NLRP3 inflammasome activation; and (iv) improve macrophage efferocytosis. These effects parallel the histological improvements observed in ESSENCE and suggest that macrophage modulation may contribute to the therapeutic effects, rather than being merely an epiphenomenon of metabolic improvement.

### 5.7 Emerging pharmacotherapies in advanced clinical development

Beyond the two FDA accelerated approval agents, multiple drug classes are in Phase 2–3 development, with varying degrees of evidence for macrophage-mediated therapeutic benefits.

#### 5.7.1 Tirzepatide (dual GIP/GLP-1 receptor agonist)

The Phase 2 SYNERGY-NASH trial demonstrated that tirzepatide 15 mg achieved MASH resolution in 62% of patients and fibrosis improvement in 51% at 72 weeks, compared to 10% and 30% on placebo, respectively (162). Given its dual incretin action, tirzepatide likely exerts macrophage-modulatory effects similar to or exceeding those of semaglutide, through both GLP-1 receptor-mediated anti-inflammatory signaling and enhanced metabolic correction. Dedicated mechanistic studies to characterize direct macrophage effects are pending.

#### 5.7.2 Survodutide (GLP-1/glucagon receptor dual agonist)

Survodutide has shown promising Phase 2 results with significant histological improvement in MASH (163). The addition of glucagon receptor agonism may enhance hepatic fat oxidation and metabolic flux, indirectly reducing macrophage-activating lipotoxic metabolites.

#### 5.7.3 Pemvidutide (GLP-1/glucagon receptor dual agonist)

Pemvidutide has emerged with early histologic efficacy, meaningful weight loss, and sustained antifibrotic signals as it advances toward Phase 3 development. As a dual-agonist targeting multiple incretin pathways, it likely combines the macrophage-modulatory effects of GLP-1 receptor activation with additional metabolic benefits through glucagon receptor signaling.

#### 5.7.4 Efruxifermin (FGF21 analog)

FGF21 analogs represent another emerging class with metabolic and anti-inflammatory properties. Longer-term data from the HARMONY and SYMMETRY trials suggest fibrosis benefits despite mixed primary results, with potential macrophage-modulatory effects through metabolic improvement and direct anti-inflammatory signaling. FGF21 receptors are expressed on multiple liver cell types, including macrophages, providing a basis for direct cellular effects.

### 5.8 Macrophage-targeted and cell-based therapies

Macrophage adoptive transfer and targeted drug delivery represent emerging strategies for directly modulating hepatic macrophage function in fibrosis, with preclinical evidence supporting the therapeutic potential of engineered anti-inflammatory macrophages (138).

#### 5.8.1 Regenerative macrophage therapy

Resolution Therapeutics’ autologous macrophage therapy represents the most direct macrophage-centered approach. In human Phase 2/3 clinical trials (AUTOSTEM, REALISTIC), data presented at AASLD 2024 and 2025 demonstrated that RMT produced early measurable improvements in liver function (international normalized ratio, model for end-stage liver disease score at 90 days), which correlated with improved long-term survival, reduced all-cause mortality, and increased transplant-free survival at three years.

Preclinical data with engineered macrophages (RTX001) expressing IL-10 and MMP9 showed significantly greater anti-inflammatory effects compared to non-engineered macrophages in CDAHFD mouse models of liver fibrosis. This demonstrates that macrophage-mediated benefits can be enhanced through genetic engineering to optimize their anti-inflammatory and anti-fibrotic phenotypes.

#### 5.8.2 Cell therapy: metabolic phenotype as a determinant of therapeutic potential

An emerging therapeutic approach for MASLD involves the adoptive transfer of ex vivo manipulated macrophages with defined metabolic phenotypes (95). This cell therapy strategy leverages the principle that metabolic phenotype determines functional properties, allowing the engineering of macrophages with optimized therapeutic characteristics.

Preclinical studies have demonstrated the feasibility of this approach. Such studies suggest that adoptive transfer of macrophages with an anti-inflammatory phenotype may ameliorate hepatic inflammation and fibrosis. The therapeutic effects of these cells persist even after the transferred cells are no longer detectable, suggesting that they exert their effects by modulating the hepatic microenvironment and potentially reprogramming endogenous macrophages (164).

The success of adoptive macrophage transfer depends critically on the metabolic state of the transferred cells. Cells that are metabolically programmed for OXPHOS and FAO are more effective at suppressing inflammation and promoting tissue repair than cells with glycolytic metabolism (144). This finding validates the principle that metabolic phenotype determines therapeutic potential and suggests that metabolic engineering should be a key component of macrophage cell therapy strategies.

Several challenges must be addressed before adoptive macrophage therapy can be translated to clinical practice. The optimal source of macrophages for ex vivo manipulation, whether autologous monocytes, induced pluripotent stem cells, or established cell lines, remains to be determined.

From a manufacturing perspective, the production of good manufacturing practice-grade macrophages faces substantial constraints, including the high costs associated with large-scale monocyte differentiation under strictly controlled conditions, the need for serum-free or chemically defined media to minimize batch-to-batch variability, and the complexity of maintaining phenotypic stability throughout the manufacturing process (165, 166). Scaling up the differentiation process for clinical application while maintaining consistent quality across batches presents a formidable obstacle, as any inconsistency in cell phenotype, purity, or function can lead to variable therapeutic outcomes (167).

Furthermore, metabolic drift represents a critical post-manufacturing challenge. Cryopreservation introduces a time-dependent metabolic artefact that favors glycolysis and impairs OXPHOS, as demonstrated in hMDMs and dendritic cells (168). In the context of macrophage cell therapy, frozen monocyte-derived macrophages exhibit significantly reduced viability (45.5% vs 75.2% in fresh macrophages) and a less advantageous phenotype, with decreased expression of anti-inflammatory markers CD206 and CD163 and increased expression of CCR2, indicating a shift toward a more pro-inflammatory classically activated macrophage-like state (165). This metabolic and phenotypic drift compromises the anti-inflammatory and tissue-repair capacities required for therapeutic efficacy upon transfer.

In the context of allogeneic therapies, human leukocyte antigen (HLA) matching presents an additional hurdle. HLA-mismatched allogeneic cells induce increased alloreactive T/B cell responses and reduced viability compared to HLA-matched cells, as demonstrated in human iPSC-derived macrophage co-culture systems with allogeneic lymphocytes (169). Although HLA-matched allogeneic cells can minimize immunogenicity, the establishment of national/international cell banking systems is required for practical implementation. Alternatively, hypoimmunogenic universal iPS cells generated through HLA-type gene knockout (e.g., HLA-A, HLA-B, and HLA-DRA deletion) offer a promising strategy to circumvent HLA mismatch risks, though the safety of such genome-edited cells, including potential tumorigenicity due to deficient antigen recognition, remains to be validated (170).

Additionally, the safety of adoptively transferred macrophages, including their potential for off-tissue activation or malignant transformation, must be carefully evaluated.

### 5.9 Combination strategies and systems approaches

The complexity of macrophage metabolic reprogramming in MASLD, with its multiple interconnected pathways and feedback loops, suggests that single-agent therapies may have limited efficacy (160). Combination approaches that target multiple nodes of the metabolic network simultaneously may be necessary to achieve durable metabolic reprogramming and disease modification (160).

Several rational combination strategies can be envisioned. Combining PPAR agonists, which restore FAO, with direct glycolytic inhibitors could simultaneously promote oxidative metabolism while suppressing glycolysis, achieving a more complete metabolic shift than either approach alone (143). Adding agents that enhance mitochondrial quality control, such as mitophagy inducers or mitochondrial biogenesis activators, could further improve the function of reprogrammed macrophages (100).

Another promising approach involves combining metabolic modulators with interventions that target the circadian regulation of metabolism (171). The circadian clock governs the rhythmic expression of metabolic genes in macrophages, and disruption of circadian rhythms has been implicated in metabolic disease (172). Time-restricted feeding or pharmacological modulation of clock genes could potentially enhance the efficacy of metabolic therapies by aligning treatment with the natural metabolic rhythms of macrophages (173).

### 5.10 Single-cell metabolomics: mapping metabolic heterogeneity

The hepatic macrophage compartment in MASLD exhibits substantial metabolic heterogeneity that bulk tissue metabolomics cannot capture. Single-cell metabolomics, combining mass spectrometry imaging with single-cell isolation techniques or metabolic flow cytometry, now enables the profiling of metabolic states in individual macrophages within their native tissue context. Recent applications of single-cell RNA-seq have already revealed distinct macrophage subpopulations, including TREM2^+^ LAMs, pro-inflammatory MoMFs, and metabolically quiescent resident KCs, each with unique transcriptional signatures suggesting divergent metabolic programs (31).

Direct metabolomic profiling at single-cell resolution has been achieved through several technical platforms. Single-cell mass spectrometry using surface-enhanced Raman scattering-microfluidic platforms has enabled the simultaneous, label-free detection of pyruvate, ATP, and lactate at the single-cell level, revealing metabolic heterogeneity related to cell density and intercellular interactions (174). Similarly, dynamic single-cell metabolomics systems based on isotope tracing and organic mass cytometry have quantified glutathione and phospholipid levels in individual cells, allowing the reconstruction of metabolic pathway interconnections at single-cell resolution (175).

In the context of liver disease, spatially resolved multi-omics has provided concrete evidence for metabolic reprogramming across disease progression. A recent integrative study combining single-cell RNA-seq, Visium spatial transcriptomics, and MALDI-MSI on 61 human livers across MASLD stages identified disease-specific spatial metabolic modules, with Module 5 (enriched in triglycerides and lipids associated with fat accumulation) exhibiting the strongest correlation with disease severity and marked upregulation in pericentral regions (176). Complementary spatial transcriptomic analysis revealed zonation-dependent gene expression patterns, with portal, periportal, mid, and central zones annotated based on established lobule markers (176). These data support the concept that hypoxic pericentral microenvironments impose distinct metabolic programs on resident cells.

Technological integration is advancing rapidly. The combination of spatial transcriptomics (Visium, multiplexed error-robust fluorescence *in situ* hybridization) with mass spectrometry imaging has generated multimodal atlases of the human liver, mapping cellular distribution and metabolic states across disease progression. For example, MERFISH analysis of fibrotic human liver identified two macrophage populations with distinct spatial distributions: Mac 2 cells (MARCO^+^, CD5L^+^, CD68^+^) distributed diffusely through the lobule, and Mac 1 cells enriched in portal areas, with fibrotic injury driving a shift toward the Mac 1 phenotype (177). The integration of these spatial transcriptomic data with MALDI-MSI metabolomic profiles has enabled the construction of comprehensive spatial atlases, providing a resource for understanding the metabolic microenvironments that shape macrophage function in MASLD.

Systems biology approaches, including metabolomics, transcriptomics, and proteomics, can help identify optimal combination strategies by mapping the metabolic network and predicting the effects of interventions (178). Single-cell technologies are particularly valuable in this regard, as they can reveal metabolic heterogeneity within the hepatic macrophage compartment and identify subpopulations that may be particularly responsive to specific interventions (31).

### 5.11 Clinical translation and therapeutic outlook

The MASLD/MASH therapeutic landscape has evolved rapidly from 2024 to 2026, with two FDA-approved agents (resmetirom, semaglutide) and multiple agents in Phase 3 development. Macrophage-mediated mechanisms contribute to therapeutic efficacy across drug classes, ranging from indirect effects via metabolic improvement (resmetirom) to direct immunomodulation (semaglutide, GLP-1 RAs) to explicit macrophage reprogramming (lanifibranor) and cell-based macrophage therapy (RMT). Future precision medicine approaches may stratify patients based on their macrophage-inflammatory phenotypes to optimize therapeutic selection.

## 6 Conclusion and future perspectives

The metabolic reprogramming of hepatic macrophages in MASLD represents a fundamental driver of disease progression, not merely a secondary consequence of inflammation. The shift from oxidative metabolism to aerobic glycolysis, enforced by HIF-1α stabilization and reinforced by epigenetic modifications, creates a self-sustaining cycle of inflammatory activation and tissue damage (8). The disruption of the TCA cycle at critical nodes generates signaling metabolites that further amplify inflammation. The IRG1-itaconate axis undergoes a dynamic U-shaped trajectory during MASLD progression: an initial depletion during simple steatosis (due to Kupffer cell loss) is followed by a marked compensatory upregulation during MASH, driven by IRG1 induction in infiltrating monocyte-derived macrophages (109 ± 9 μM in human NASH livers; 400 ± 131 μM in WD-fed mouse livers). However, counter-regulatory β-arrestin 2-mediated IRG1 degradation limits the magnitude of this response, preventing full anti-inflammatory resolution and perpetuating the glycolytic-inflammatory cycle (14). The direction and cell-type-specific regulation of itaconate across MASLD stages are now partially defined, though the precise functional thresholds for therapeutic intervention require further investigation.

The failure of repair programs mediated by TREM2^+^ LAMs compounds these metabolic derangements. ADAM17-mediated TREM2 shedding impairs efferocytosis, leading to the accumulation of uncleared apoptotic cells and perpetuating DAMP-driven inflammatory signaling (31). The resulting vicious cycle of metabolic dysfunction, inflammation, and tissue damage drives the progression from simple steatosis to steatohepatitis, fibrosis, and ultimately cirrhosis. Notably, while the IRG1-itaconate axis mounts a compensatory anti-inflammatory response in MASH, the concurrent β-arrestin 2-mediated IRG1 degradation and metabolic exhaustion of LAMs prevent this response from achieving therapeutic efficacy, highlighting the need for exogenous itaconate augmentation or β-arrestin 2 inhibition as complementary therapeutic strategies.

Encouragingly, emerging evidence suggests that these metabolic states are not fixed but exhibit considerable plasticity. PPAR agonists, direct glycolytic inhibitors, and itaconate analogs have all shown promise in preclinical models, demonstrating that metabolic reprogramming can be therapeutically targeted.

The field is transitioning from merely documenting metabolic alterations to testing whether reversing these changes can modify disease outcomes. This shift requires the development of better tools for assessing macrophage metabolism *in vivo*, including metabolic imaging techniques and biomarkers that reflect the metabolic state of hepatic macrophages (178). Single-cell metabolomics, which can profile the metabolic state of individual cells within complex tissues, holds particular promise for identifying macrophage subpopulations that are most amenable to metabolic intervention (31).

Looking forward, several key challenges and opportunities can be identified. First, the development of combination therapies that target multiple nodes of the metabolic network simultaneously may achieve more durable metabolic reprogramming than single-agent approaches (160). Rational combinations based on systems-level understanding of metabolic networks, rather than empirical trial and error, will be essential for optimizing therapeutic outcomes.

Second, the identification of metabolic state biomarkers that can predict treatment response will be critical for implementing precision medicine approaches in MASLD (178). Promising candidate biomarkers reflecting macrophage metabolic states include: (i) the succinate-to-fumarate ratio, which indicates SDH breakpoint activity and HIF-1α stabilization potential; (ii) the lactate-to-pyruvate ratio, a readout of glycolytic commitment and cellular redox state; (iii) circulating itaconate levels, which may reflect IRG1 activity and anti-inflammatory metabolic reserve; (iv) acylcarnitine profiles, particularly long-chain species (C16:0, C18:0), as indicators of impaired mitochondrial β-oxidation; and (v) the NAD^+^/NADH ratio, assessable through plasma nicotinamide metabolite panels, as a surrogate for cellular oxidative capacity. Not all patients are likely to respond equally to metabolic therapies, and biomarkers that identify responders versus non-responders could guide treatment selection and improve clinical trial efficiency.

Third, the timing of metabolic interventions may be crucial. Metabolic plasticity may decline as disease progresses and epigenetic modifications become more entrenched (136). Early intervention, before the establishment of self-reinforcing metabolic cycles and irreversible structural changes, may be necessary to achieve optimal therapeutic outcomes.

Finally, the integration of metabolic therapies with other treatment modalities, including lifestyle interventions, bariatric surgery, and emerging therapies targeting hepatocyte lipid metabolism or hepatic stellate cell activation, will be important for comprehensive MASLD management (160). Macrophage metabolism does not operate in isolation but is intimately connected to the metabolic state of other liver cell types, and systemic metabolic improvements may be necessary for optimal therapeutic effects.

In conclusion, the metabolic reprogramming of hepatic macrophages in MASLD represents both a fundamental disease mechanism and a promising therapeutic target. The opportunity to fundamentally alter disease trajectory by repairing cellular metabolism is real and increasingly within reach. As our understanding of macrophage immunometabolism continues to advance and therapeutic tools become more sophisticated, the prospect of metabolic therapies that restore hepatic macrophage homeostasis and halt MASLD progression is becoming increasingly tangible (179).

## Glossary

4-OI: 4-octyl itaconate
ACC: acetyl-CoA carboxylase
ACL: ATP-citrate lyase
ADAM17: ADAM metallopeptidase domain 17
ATP: adenosine triphosphate
BA: bile acid
CCL2: C-C motif chemokine ligand 2
CCR2: C-C chemokine receptor type 2
CCR5: C-C chemokine receptor type 5
CD36: cluster of differentiation 36
CDAHFD: choline-deficient, L-amino acid-defined, high-fat diet
CPT1: carnitine palmitoyltransferase 1
DAMPs: damage-associated molecular patterns
DI: dimethyl itaconate
ETC: electron transport chain
FAO: fatty acid oxidation
FAD: flavin adenine dinucleotide
FGF21: fibroblast growth factor 21
FXR: farnesoid X receptor
GIP: glucose-dependent insulinotropic polypeptide
GLP-1: glucagon-like peptide-1
GLUT: glucose transporter
GPR91: G protein-coupled receptor 91
HCC: hepatocellular carcinoma
HFD: high-fat diet
HIF-1α: hypoxia-inducible factor-1α
HK2: hexokinase 2
HLA: human leukocyte antigen
IL: interleukin
IL-1β: interleukin-1 beta
IL-6: interleukin-6
IRG1: immune-responsive gene 1
IκBζ: inhibitor of kappa B zeta
KC: Kupffer cell
LAM: lipid-associated macrophage
M2: alternatively activated macrophage
MASH: metabolic dysfunction-associated steatohepatitis
MASLD: metabolic dysfunction-associated steatotic liver disease
mtROS: mitochondrial reactive oxygen species
NADH: reduced nicotinamide adenine dinucleotide
NASH: non-alcoholic steatohepatitis
NF-κB: nuclear factor kappa-light-chain-enhancer of activated B cells
NLRP3: NOD-like receptor protein 3
Nrf2: nuclear factor erythroid 2-related factor 2
OXPHOS: oxidative phosphorylation
PDH: pyruvate dehydrogenase
PDK: pyruvate dehydrogenase kinase
PHD: prolyl hydroxylase
PKM2: pyruvate kinase M2
PPAR: peroxisome proliferator-activated receptor
PPARα: peroxisome proliferator-activated receptor alpha
RMT: regenerative macrophage therapy
ROS: reactive oxygen species
SAM: scar-associated macrophage
SCFAs: short-chain fatty acids
SDH: succinate dehydrogenase
SUCNR1: succinate receptor 1
TCA: tricarboxylic acid
TGF: transforming growth factor
TGR5: Takeda G protein-coupled receptor 5
THP-1: tohoku hospital pediatrics-1
TLR4: Toll-like receptor 4
TNF-α: tumor necrosis factor-alpha
TREM2: triggering receptor expressed on myeloid cells 2
